# Enhancing Osteogenesis in Osteoporosis via Electromagnetized Gold Nanoparticles

**DOI:** 10.34133/bmr.0260

**Published:** 2025-09-24

**Authors:** Yang Liu, Yan Li, Xue Bai, Yu Gu

**Affiliations:** School of Biomedical Engineering, Capital Medical University, 100069 Beijing, China.

## Abstract

Osteoporosis (OP) is the most common bone metabolic disorder worldwide, markedly compromising patients’ quality of life and imposing a substantial healthcare burden. However, current clinical treatments for OP are not able to provide satisfactory therapeutic outcomes, particularly in the presence of complex inflammatory conditions. The integration of noninvasive physical therapy and bionanotechnology has shown great promise in modulating cellular functions and optimizing the bone microenvironment. In this study, we demonstrated that electromagnetized gold nanoparticles (AuNPs) exhibited excellent biocompatibility at the cellular, vascular, and major organ levels. These electromagnetized AuNPs significantly enhanced the biological behaviors of osteoblasts, including proliferation, migration, colony formation, and osteogenic differentiation. Remarkably, RNA sequencing analysis revealed that electromagnetized AuNPs significantly activated the mitochondrial oxidative phosphorylation pathway while suppressing the interleukin-17 pro-inflammatory signaling pathway. Additionally, electromagnetized AuNPs stabilized mitochondrial membrane potential and boosted adenosine triphosphate (ATP) production while reducing cell apoptosis and oxidative stress, thereby promoting osteogenic differentiation under inflammatory conditions. Furthermore, in a mouse model of inflammation-induced OP, the electromagnetized AuNPs effectively restored bone mass and improved trabecular architecture. Collectively, our findings provide a proof-of-concept that electromagnetized AuNPs enhance osteogenesis by promoting osteogenic differentiation and optimizing the bone microenvironment, highlighting their potential as a promising therapeutic strategy for OP.

## Introduction

Osteoporosis (OP) is a systemic metabolic disorder characterized by reduced bone mass and deteriorated bone microstructure, leading to increased bone fragility and susceptibility to fractures [1]. With the accelerating global aging population, the prevalence of OP is steadily increasing each year. Projections suggest that by 2050, over 400 million individuals worldwide will be affected by this condition, posing a substantial public health challenge [2]. The currently available treatments for OP predominantly rely on pharmacological interventions, including anti-resorptive and bone anabolic agents. Anti-resorptive drugs, such as estrogen [3], bisphosphonates [4], and calcitonin [5], effectively inhibit bone resorption, preventing further bone loss and maintaining skeletal stability. However, these agents are unable to reverse existing bone loss and improve bone microarchitecture. By comparison, parathyroid hormone (PTH) analogs (e.g., teriparatide) and sclerostin inhibitors (e.g., romosozumab) promote new bone formation as anabolic metabolic agents, yet their prolonged use may accelerate bone resorption, leading to instability in therapeutic outcomes [6]. Moreover, the existing pharmacological treatments are associated with a range of severe adverse effects, including abnormal uterine bleeding [7], increased cardiovascular risk [8], osteonecrosis of the jaw [9], atypical femoral fractures [10], and even osteosarcoma [11]. For these reasons, developing a safe and effective therapeutic strategy for OP remains a pressing clinical need.

Osteoblasts play a pivotal role in bone remodeling, with their biological functions and osteogenic differentiation potential directly determining the efficiency of bone formation and the maintenance of bone mass [12]. Under osteoporotic conditions, the biological functions of osteoblasts are severely impaired, characterized by reduced proliferation, migration, and osteogenic differentiation capacity, leading to decreased synthesis of bone matrix, insufficient mineralization, and ultimately bone loss and microstructural deterioration [13]. Consequently, enhancing osteoblast activity and differentiation potential has become a key therapeutic strategy for OP. Notably, bone metabolism is intricately regulated by multiple factors, among which microenvironmental homeostasis exerts a profound influence on osteoblast function and status. Accumulating evidence indicates that OP is frequently associated with microenvironmental dysregulation, wherein inflammatory signaling serves as a critical factor in disrupting osteoblast differentiation and functional maintenance [14–17]. Specifically, pro-inflammatory cytokines such as tumor necrosis factor-α (TNF-α), interleukin-1β (IL-1β), and IL-6 have been shown to modulate osteogenic signaling pathways and alter cellular responsiveness to external stimuli [18]. Moreover, elevated oxidative stress further exacerbates the disruption of osteoblast homeostasis [19]. Thus, while augmenting osteoblast function to promote bone formation, maintaining a favorable microenvironment is essential for the effective management of OP.

The discovery of the piezoelectric properties of bone has established a biophysical foundation for utilizing exogenous electromagnetic signals to regulate bone cell biology [20]. As a noninvasive physical therapy, electromagnetic field (EMF) has been demonstrated to enhance osteoblast growth and differentiation, thereby facilitating osteogenesis through the generation of weak electric currents and the modulation of ion distribution [21–23]. However, its application in OP treatment still faces several challenges. On the one hand, due to the dielectric properties of biological tissues, electromagnetic signals are susceptible to energy attenuation and scattering during propagation [24–26], making it difficult to maintain sufficient field strength in deep bone tissues, particularly in fracture-prone regions such as the hip and spine [27]. On the other hand, within the complex OP microenvironment, the elevated expression of inflammatory cytokines and oxidative stress molecules may interfere with the regulatory effects of electromagnetic signals on osteoblast cell functions, thereby diminishing the biological efficacy of EMF [28,29].

With the continuous advancement of bionanotechnology, the combined therapeutic strategy of physical stimulation and nanoparticles holds great promise in overcoming the aforementioned limitations. Firstly, nanoparticles can enter cells via endocytosis, enhancing cellular responsiveness to physical stimuli and amplifying the intensity of localized physical fields, thereby optimizing signal transmission efficiency [30]. Meanwhile, nanoparticles exhibit strong interactions with biological systems, modulating the local microenvironment to enhance cellular biological responses [31]. Of these, gold nanoparticles (AuNPs) have garnered widespread attention due to their ease of functionalization, excellent biocompatibility, and anti-inflammatory properties [32,33]. Furthermore, AuNPs have been shown to act as distributed nanoelectrodes under EMF, thereby locally enhancing the electric field strength near the plasma membrane and further improving the penetration efficiency of EMF [34]. Additionally, AuNPs exposed to specific EMF frequencies become transiently magnetized through the “Fermi hole effect”, enabling a more efficient transfer of electromagnetic energy to target cells [35,36]. Our previous studies have demonstrated that AuNPs, as therapeutic agents, can mitigate inflammation, thereby ameliorating excessive bone loss in inflammatory bone diseases [37–39]. Based on these findings, we hypothesize that the combination of AuNPs and EMF may exert synergistic effects in enhancing the biological functions and osteogenic differentiation of osteoblasts while simultaneously modulating and optimizing the bone microenvironment, thereby promoting osteogenesis under osteoporotic conditions.

**Fig. 1.**
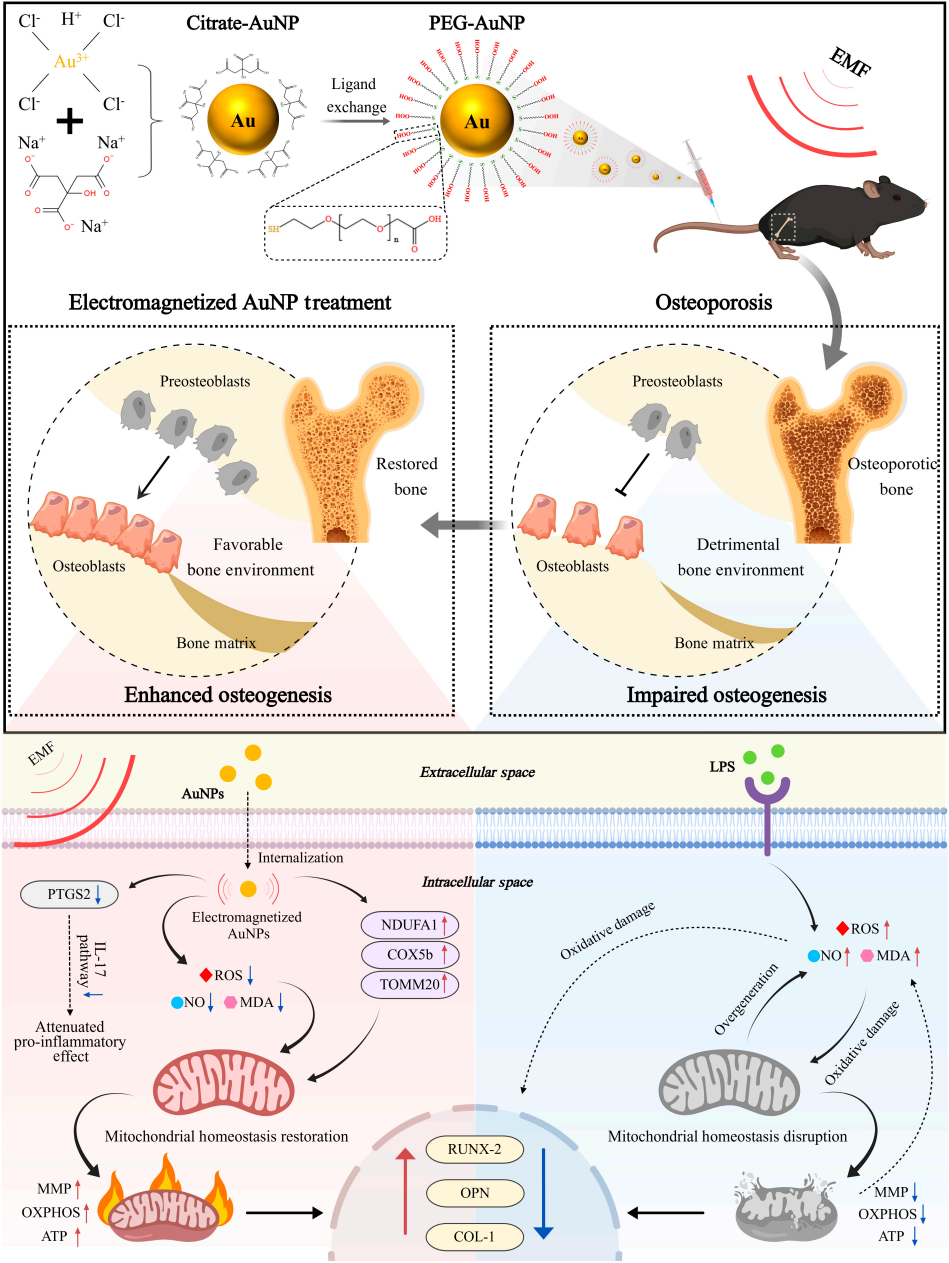
Schematic illustration of osteogenesis enhancement in OP using electromagnetized AuNPs. PEGylated AuNPs were administered to experimental mice and exposed to an EMF of specific frequency and intensity. These AuNPs exhibit electromagnetic responsiveness, enabling the efficient transfer of electromagnetic stimulation to target cells. By stabilizing mitochondrial homeostasis, electromagnetized AuNPs promote osteogenic differentiation and contribute to a favorable bone microenvironment, thereby enhancing osteogenesis and mitigating OP.

In this study, we demonstrated the potential of using electromagnetized AuNPs for OP treatment (Fig. 1). These AuNPs were activated and electromagnetized under a specific frequency and intensity of EMF, enabling a more direct transfer of electromagnetic stimulation to target cells. To evaluate the biocompatibility of electromagnetized AuNPs both in vitro and in vivo, we systematically examined their effects on cell viability, hemolysis, and histopathological changes in major organs. Moreover, we found that electromagnetized AuNPs exhibited superior osteogenic effects compared to individual nanoparticles or physical stimulation alone. Mechanistic investigations revealed that electromagnetized AuNPs could significantly up-regulate genes and proteins associated with mitochondrial function, thereby enhancing oxidative phosphorylation (OXPHOS) and energy production. Concurrently, they inhibited the IL-17 pro-inflammatory signaling pathway, reducing inflammation and oxidative stress while maintaining a favorable bone microenvironment. Further in vitro experiments confirmed that electromagnetized AuNPs alleviated mitochondrial membrane potential (MMP) disruption and adenosine triphosphate (ATP) reduction induced by inflammatory stimulation while simultaneously reducing apoptosis and modulating oxidative stress levels, thereby promoting in vitro osteogenesis. Additionally, we evaluated the therapeutic efficacy of electromagnetized AuNPs in an inflammation-induced osteoporotic mouse model. As predicted, electromagnetized AuNPs effectively mitigated inflammation-induced impairment and suppression of bone formation, restored bone mass, and improved bone microstructure. Therefore, our findings indicated that electromagnetized AuNPs are expected to promote osteogenic differentiation and optimize the bone microenvironment, offering a potentially viable and safe treatment strategy for OP.

## Materials and Methods

### Synthesis of AuNPs

Citrate-coated AuNPs (citrate-AuNPs) were synthesized from HAuCl_4_ by using the classic citrate reduction method, as reported previously [40]. Specifically, a 1% HAuCl_4_ solution was diluted 100-fold with ultrapure water and heated to boiling, maintaining a pH of 3.42 to 3.46. Subsequently, a 5% sodium citrate solution was added to the HAuCl_4_ solution in a 1:2 volume ratio. The mixture was heated until it developed a wine-red color, indicating AuNP formation, with no further color change. After cooling the solution to the room temperature, it was centrifuged at 14,000*g* for 20 min to separate the nanoparticles. The supernatant was discarded, and the precipitate was resuspended in ultrapure water. To enhance stability, polyethylene glycol (PEG) was added under a nitrogen atmosphere, and the solution was stirred overnight in a sealed tube. Finally, the resulting solution was centrifuged again (14,000*g*, 20 min), the supernatant was discarded, and the PEGylated AuNP (PEG-AuNPs) precipitate was redispersed in ultrapure water for future experimental use.

### Characterization of AuNPs

The synthesized AuNPs were characterized for their physicochemical properties. The surface plasmon resonance absorption spectrum of the AuNPs was measured using an ultraviolet and visible (UV–vis) spectrophotometer (UV3600, Shimadzu, Japan) to confirm nanoparticle formation and size distribution. Hydrodynamic diameter and ζ potential were analyzed through dynamic light scattering (DLS) (Zetasizer Nano ZS90, Malvern Panalytical, UK). Structural morphology and elemental composition of the AuNPs were examined through transmission electron microscopy (TEM) (Tecnai G2 F30 S-TWIN, Field Electron and Ion Company, USA) and x-ray diffraction (XRD) (Bruker D8 Advance, Bruker AXS, Germany). The elemental composition and concentration of the AuNPs were quantified through inductively coupled plasma mass spectrometry (ICP-MS) (710, Agilent, USA).

### EMF exposure

An electromagnetic generation system was developed, incorporating a power amplifier, signal generator, oscilloscope, and Helmholtz coils. The coil configuration was designed using COMSOL Multiphysics software; it consisted of 2 identical circular magnetic coils that created an almost highly uniform magnetic field within the central region. For in vitro applications, the outer diameter of the coils is 220 mm, the inner diameter is 150 mm, and the distance between the 2 coils is 110 mm. To accommodate the freely moving state of the mice and ensure adequate EMF coverage, the in vivo coil system was specifically designed with a larger inner diameter and wider spacing. For in vivo EMF exposure, the outer diameter, inner diameter, and distance between the 2 coils are 265, 190, and 132.5 mm, respectively. The in vitro coil configuration comprises 80 turns of enameled copper wire with a cross-sectional dimension of 1.6 × 3 mm. The in vivo coil configuration includes 190 turns of enameled copper wire with a winding diameter of 2.8 mm. A signal generator (UTG9000T, UNI-T, China) was used to produce the pulsed EMF (PEMF) waveform, which was monitored and validated for both waveform and field strength by using an oscilloscope (UTD2000CEX+, UNI-T, China) and a Gauss meter (TM-701, Kanetec, Japan), respectively. The field strength measured by the Gauss meter represents the effective field strength ($\bar{X}$) of PEMF. The peak field strength (*X*) needs to be calculated based on the waveform and pulse frequency:

$$\bar{X}=\int_{0}^{x}\frac{X\times t\times s}{T}$$ (1)

where *T* is the period, *t* is the pulse width, and *s* is the duty cycle of the pulse train. Cell culture plates for in vitro experiments and mouse cages for in vivo studies were positioned precisely at the midpoint between the coils to ensure uniform exposure (Fig. S1). Another pair of Helmholtz coils, which is not connected to the signal generator, served as a control (sham exposure group). Throughout the experiment, the temperature was maintained at 37 ± 0.1 °C to eliminate the potential thermal effects during cultivation.

### Cell culture

The MC3T3-E1 mouse pre-osteoblast cell line was used in this study. These cells were sourced from the National Laboratory Cell Resource Sharing Platform and verified for authenticity by performing short tandem repeat analysis. Routine mycoplasma testing was performed every 3 months using the MycoSensor PCR Assay Kit (Agilent) to confirm cell line purity. After thawing, the cells were seeded in 10-cm culture dishes and incubated at 37 °C in a humidified atmosphere with 5% CO_2_. The culture medium consisted of MEM Alpha (Gibco, USA), supplemented with 10% fetal bovine serum (Fetal Bovine Serum Premium Plus, Gibco), 1% penicillin–streptomycin solution (Gibco), and 3 mM l-glutamine (Gibco). Lipopolysaccharide (LPS; 1,000 ng/ml) was used to establish an in vitro inflammatory microenvironment.

### Cell viability

Cell viability was assessed by using the Cell Counting Kit-8 (CCK-8) kit as per the standard protocols. Briefly, each treatment group was incubated with a culture medium containing 10% CCK-8 solution (96992 Cell Counting Kit-8, Sigma-Aldrich, USA) for 2 h. Absorbance was then measured at 450 nm with a microplate reader (SpectraMax i3x, Molecular Devices, USA).

### Live/dead cell staining

The experimental cells were seeded in 12-well plates and washed once with phosphate-buffered saline (PBS) following various treatments. The working solution (200 μl) containing calcein AM and ethidium homodimer-1 (LIVE/DEAD, Invitrogen, USA) was added to each well and incubated in the dark at room temperature for 30 min. Finally, fluorescent images were captured by using an inverted fluorescence microscope (ECLIPSE Ts2R, Nikon, Japan) at excitation/emission wavelengths of 494/571 nm and 528/617 nm, respectively.

### Internalization of AuNPs

After seeding cells into a 6-well plate and allowing cell adhesion, AuNPs were added and incubated for 24 h. Half of the cell samples were harvested by using a cell scraper, fixed in 2.5% glutaraldehyde at 4 °C for 24 h, and then subjected to secondary fixation with 1% OsO_4_ for 90 min. Following 3 phosphate buffered rinses (0.1 M, pH 7.0), the samples were dehydrated through an ethanol graded series (30%, 50%, 70%, 80%, 90%, and 100%, each for 15 min) and then treated with pure acetone for 20 min. Then, infiltration was performed by using a graded mixture of acetone and resin (3:1 for 2 h, 1:1 for 3 h, and 1:3 for 3 h), with an overnight incubation in pure resin. The samples were then embedded in molds and subjected to gradient heating (35, 60, and 80 °C for 5 h each). Ultrathin sections (70 to 90 nm) were prepared using an ultramicrotome (EM UC7, LEICA, Germany), stained with uranyl acetate (15 min) and lead citrate (5 min), air-dried, and visualized through TEM (JEM1200EX, JEOL, Japan). For quantitative analysis, the remaining cells were trypsinized and counted. Then, 1 ml of the cell suspension was mixed with 3 ml of aqua regia, heated at 200 °C for 30 min for digestion, cooled, and diluted to a final volume for ICP-MS analysis (710, Agilent) to determine the Au concentration. To evaluate the accumulation of AuNPs in bone tissue following systemic administration, mice were intravenously injected with PEG-AuNPs at a dose of 60 μg/kg. At 6 and 24 h post-injection, bilateral hindlimb bones were harvested, rinsed with PBS, dried, and digested in aqua regia under the same conditions. After dilution, the gold content was determined by ICP-MS and expressed as ng of Au per gram of bone tissue (ng/g).

### Hemolytic experiment

To assess the hemolytic potential, blood was collected from the apex of healthy mouse hearts, and fibrinogen was removed. Blood samples were diluted in approximately 10 times the volume of 0.9% NaCl solution, mixed thoroughly, and centrifuged at 1,500 rpm for 15 min. The supernatant was removed, and the cells were washed thrice until the supernatant became clear and colorless. A 4% red blood cell (RBC) suspension was prepared, and various AuNP concentrations (0, 0.12, 0.6, 3, 15, 75, and 375 μg/ml) were added and incubated with gentle mixing. Varying Triton X-100 concentrations (0%, 0.00016%, 0.008%, 0.04%, 0.2%, 1%, and 5%) were used as positive controls. The samples were incubated at 37 °C for 1 h and centrifuged at 1,000*g* for 3 min. Hemolysis was indicated by a clear red supernatant with minimal cell pellet, while a clear supernatant with a pellet indicated no hemolysis. Each supernatant (100 μl) was transferred into a 96-well plate, and the absorbance was measured at 540 nm by using the microplate reader (SpectraMax i3x, Molecular Devices, USA). The hemolysis rate for each treatment group was calculated as follows: Hemolysis rate = (OD sample − OD negative control)/(OD positive control − OD negative control).

### Cell proliferation assay

Cell proliferation was assessed by using the Click-iT EdU Imaging Kits (Invitrogen) following the manufacturer’s protocol. Briefly, the cells treated from each experimental group were incubated with a pre-warmed 2× EdU (5-ethynyl-2′-deoxyuridine) working solution (20 μM) at 37 °C for 2 h to allow EdU incorporation into newly synthesized DNA. After incubation, the cells were fixed with 4% paraformaldehyde at room temperature for 15 min, washed thrice, and permeabilized with PBS containing 0.5% Triton X-100 for 15 min. Following 2 additional washes, 200 μl of Click reaction cocktail was added, and the cells were incubated in the dark at room temperature for 30 min. The cells were subsequently stained with 1× Hoechst for nuclear visualization, washed thrice, and immediately subjected to fluorescence microscopy. The percentage of EdU-positive cells was quantified by using ImageJ software (National Institutes of Health, USA) at excitation/emission wavelengths of 346/460 nm for Hoechst and 555/565 nm for EdU.

### Clone formation assay

Cell colony formation was evaluated to determine proliferative potential after treatment. The cells were routinely digested, detached, and centrifuged to collect cell pellets. After the cells were counted, they were seeded into culture plates at a density of 1,000 cells per well in 6-well plates (7-d assay) and 500 cells per well in 12-well plates (14-d assay). After treatments corresponding to each experimental group were applied, the cultures were terminated on days 7 and 14. The cells were gently washed thrice with PBS, fixed with 4% paraformaldehyde at room temperature for 15 min, and stained with 0.1% crystal violet staining solution for 30 min. Excess stain was carefully rinsed away, and the plates were air-dried. Finally, the number of colonies (defined as clusters containing more than 50 cells) was counted and imaged under a microscope (Primovert, ZEISS, Germany). The colony formation rate was calculated as follows: Colony formation rate = (average amount of colonies/number of seeded cells) × 100%.

### Wound healing assay

To evaluate cell migration, a wound healing assay was performed. Untreated cells were seeded at a density of 5 × 10^5^ cells per well in a 6-well plate and cultured to full confluence. A vertical scratch was made across the monolayer by using a 200-μl pipette tip. After the scratch, each experimental group received the appropriate treatment. At 0, 12, 24, and 36 h after the scratch, the wound area (distance between the edges of the scratch) was imaged by using a microscope (Primovert, ZEISS). Wound closure was quantified using ImageJ software (National Institutes of Health), calculating the wound healing rate as follows: Wound healing rate = [(Wound length at 0 h − Wound length at observation time point)/Wound length at 0 h] × 100%.

### In vitro osteogenic differentiation

Alkaline phosphatase (ALP) expression was measured on days 3, 7, and 14 of osteogenic induction by using an ALP staining kit (BCIP/NBT Alkaline Phosphatase Color Development Kit, Beyotime, China) and an ALP activity assay kit (Alkaline Phosphatase Assay Kit, Beyotime). Briefly, the cells were fixed with 4% paraformaldehyde at room temperature for 15 min, washed thrice with PBS, and incubated with the bromochloroindolyl phosphate/nitro blue tetrazolium (BCIP/NBT) staining solution for 2 h in the dark at the room temperature. Images of the stained cells were captured by using an optical microscope (Primovert, ZEISS), and the ALP-positive areas were quantified using ImageJ software (National Institutes of Health). To measure the ALP activity, total protein was extracted from the treated cells by using inhibitor-free lysis buffer and quantified. Subsequently, a chromogenic substrate was added and incubated at 37 °C for 10 min. The reaction was stopped, and the absorbance was read at 405 nm. The enzyme activity was calculated based on a standard curve. On days 21 and 28 of osteogenic induction, the cells were fixed with 4% paraformaldehyde at room temperature for 15 min, washed 3 times with PBS, and incubated with Alizarin Red S (ARS) solution (Alizarin Red S Solution, OriCell, USA) at 37 °C for 30 min. The stained mineralized nodules were imaged and photographed under an optical microscope (Primovert, ZEISS), and the areas were quantified using ImageJ software (National Institutes of Health). To quantify calcium deposition, calcium nodules were dissolved in 10% cetylpyridinium chloride solution, and the absorbance of the solution was measured at 570 nm.

### Transcriptome analysis

Total RNA was isolated using the TRIzol reagent, followed by RNA integrity assessment using a 2100 Bioanalyzer (Agilent). RNA purity was measured using a NanoPhotometer (Implen GmbH, Germany). Subsequently, cDNA libraries were generated using the NEBNext Ultra RNA Library Prep Kit (New England Biolabs, USA). Following library quality control and pooling, Illumina sequencing was performed on a NovaSeq 6000 platform (Illumina, USA), producing 150–base pair paired-end reads. The sequencing reads were base-called using CASAVA, followed by quality control and alignment to the reference genome using the HISAT2 tool. The gene expression was quantified using featureCounts, and fragments per kilobase of exon per million mapped reads values were calculated based on gene length. Differentially expressed genes (DEGs) were identified using the DESeq2 package and a threshold of |log_2_​(fold change) | > 0 and *P* < 0.05. This approach was designed to capture subtle but functionally significant expression changes, particularly in tightly regulated pathways where such changes may exert cumulative effects and impact downstream processes, especially in response to mild stimuli. Subsequently, the DEGs were subjected to pathway enrichment analysis by using Gene Ontology (GO) and Kyoto Encyclopedia of Genes and Genomes (KEGG), selecting terms and pathways based on an adjusted *P*-value threshold of <0.05. In addition, Gene Set Enrichment Analysis (GSEA) was performed on the GO and KEGG datasets for mouse species. Finally, data were visualized using the online platform https://www.bioinformatics.com.cn.

### RNA isolation and quantitative reverse transcription polymerase chain reaction

Cells and femoral bone tissues were harvested after treatment, and total RNA was extracted using the QuickEasy Cell Direct RT-qPCR Kit–TaqMan (Foregene, China) following the manufacturer’s instructions. Purified RNA was then converted to complementary DNA (cDNA), which was then mixed with primers and qPCR Mix-TaqMan for real-time quantitative polymerase chain reaction (PCR) (QuantStudio6, Applied Biosystems, USA). The expression levels of OXPHOS (Ndufa1, Cox5b, Tomm20) and proinflammatory (Ptgs2, IL-17A, IL-17F, IL-17RA) genes were analyzed, with glyceraldehyde-3-phosphate dehydrogenase (GAPDH) as the housekeeping gene. Relative gene expression was determined using the 2^−∆∆Ct^ method, and all primers are listed in Table S1.

### Western blotting and immunofluorescence assays

For protein extraction, cells and femoral bone tissues were lysed with radioimmunoprecipitation assay (RIPA) lysis buffer supplemented with protease inhibitors (BL504A, Biosharp, China). Protein concentrations were determined using the BCA (bicinchoninic acid) assay (G2026-1000T, Servicebio, China). Equal amounts of protein (40 μg) were separated by 10% and 12% sodium dodecyl sulfate–polyacrylamide gel electrophoresis (SDS-PAGE) and transferred onto polyvinylidene difluoride (PVDF) membranes (IPVH00010, Millipore, USA) at a constant voltage of 100 V. After blocking at room temperature for 1 h, the membranes were incubated overnight at 4 °C with primary antibodies against cyclooxygenase-2 (COX2) (1:1,000), TOMM20 (1:1,000), COX5b (1:1,000), NDUFA1 (1:1,000), IL-17A (1:1,000), IL-17F (1:1,000), IL-17RA (1:1,000), GAPDH (1:10,000), and ACTIN (1:1,000). Later, the membranes were incubated with horseradish peroxidase (HRP)-conjugated goat anti-rabbit secondary antibody (1:3,000) for 30 min. Chemiluminescence was detected using an ECL chemiluminescence detection kit (MA0186, Meilunbio, China) and visualized using an imaging system (SCG-W2000, Servicebio). For immunofluorescence (IF) staining, cells were fixed with 4% paraformaldehyde for 15 min, permeabilized with Triton X-100 for 10 min, and blocked with 5% bovine serum albumin (BSA) for 30 min. The cells were then incubated overnight at 4 °C with primary antibodies against Ki67 (1:200), RUNX2 (1:1,000), OPN (1:1,000), and COL1 (1:1,000). After the cells were washed, they were incubated with Cy3-conjugated (excitation/emission wavelengths of 552/570 nm) goat anti-rabbit secondary antibody (1:500) for 1 h, followed by nuclear staining with 4′,6-diamidino-2-phenylindole (DAPI) (62248, Thermo Fisher Scientific, USA) and actin staining with Alexa Fluor 488 (A12379, Thermo Fisher Scientific). Fluorescence was captured using a fluorescence microscope (ECLIPSE Ts2R, Nikon, Japan). Excitation/emission wavelengths for DAPI and actin were 341/452 nm and 495/518 nm, respectively. Fluorescence intensity distribution across groups was quantified using ImageJ software (National Institutes of Health).

### Cell apoptosis assay

Cell apoptosis was evaluated using the Annexin V-FITC Apoptosis Detection Kit (BMS500FI-100, Invitrogen) according to the manufacturer’s instructions. The cells from each treatment group were harvested by digestion with trypsin without EDTA, washed twice with PBS, and resuspended in 200 μl of Binding Buffer to achieve a cell density of 5 × 10^5^ cells/ml. Subsequently, 5 μl of Annexin V-FITC (fluorescein isothiocyanate) and 10 μl of propidium iodide were added to each sample, and the mixture was incubated in the dark for 10 min. The prepared samples were then analyzed through flow cytometry by using the LSRFortessa system (BD, USA).

### Detection of MMP

MMP in the various treatment groups was assessed using the JC-1 fluorescence assay. After the treatment, the cells were incubated with 2 μM JC-1 (JC-1 Mitochondrial Membrane Potential Assay Kit, MedChemExpress, USA) at 37 °C for 20 min in a cell culture incubator, washed twice with PBS, and stained with Hoechst (Hoechst 33342 Staining Solution for Live Cells, Beyotime) to visualize and label the nuclei. The OXPHOS inhibitor carbonyl cyanide m-chlorophenylhydrazone (CCCP) was added to induce a decrease in MMP as a positive control. The cells were then visualized and imaged using a laser confocal microscope (NSR950, Nexcope, China) with excitation/emission wavelengths of 346/460 nm, 510/527 nm, and 585/590 nm for blue, green, and red fluorescence, respectively. The fluorescence intensity of the acquired images was quantified using ImageJ software (National Institutes of Health).

### Measurement of ATP content

Following multiple washes with PBS, the cells were lysed using 200 μl of lysis buffer from the ATP assay kit (Beyotime). The lysates were then centrifuged at 12,000*g* for 5 min at 4 °C, and the resulting supernatant was collected for subsequent analysis. For the assay, 100 μl of assay working solution and 20 μl of lysates were added to each well of a black 96-well plate. The luminescence intensity was then measured using a microplate reader (SpectraMax i3x, Molecular Devices).

### Evaluation of oxidative stress level

The oxidative stress levels were primarily assessed by measuring the expression of reactive oxygen species (ROS), nitric oxide (NO), and malondialdehyde (MDA). The ROS levels in the treated cells were measured using the Reactive Oxygen Species Assay Kit (Beyotime). Cells were incubated with a 10 μM DCFH-DA (2′,7′-dichlorodihydrofluorescein diacetate) fluorescent probe at 37 °C for 20 min, followed by immediate imaging under a fluorescence microscope with excitation/emission wavelengths of 488 and 525 nm, respectively. The NO levels were quantified using the classical Griess reagent method (Nitric Oxide Assay Kit, Beyotime). Culture supernatants and cell lysates were collected after the treatment. For the culture supernatants, Griess reagents I and II were directly added, followed by a 10-min incubation at room temperature in the dark. Absorbance was measured at 540 nm, and nitrite concentration was calculated against a standard curve. For intracellular NO measurement, the cells were washed with PBS, lysed using lysis buffer without protease inhibitors (Cell and Tissue Lysis Buffer for Nitric Oxide Assay, Beyotime), and subjected to the same Griess reagent protocol to determine nitrite levels. MDA levels, an indicator of lipid peroxidation, were measured using the Lipid Peroxidation MDA Assay Kit (Beyotime). Total protein was extracted from the treated cells, and protein concentrations were quantified. The samples were combined with the MDA detection reagent, heated in a 100 °C water bath for 15 min, cooled to room temperature, and centrifuged at 1,000*g* for 10 min. The absorbance of the supernatant was measured at 532 nm. MDA concentrations were then calculated based on a standard curve.

### LPS-induced OP mouse model

All animal procedures were conducted in strict compliance with institutional ethical standards and approved by the Animal Welfare Committee of Capital Medical University, following guidelines from the Association for Assessment and Accreditation of Laboratory Animal Care. Six-week-old C57BL/6 male mice were randomly assigned to 3 groups (*n* = 5/group): control, LPS, and electromagnetized AuNP. The animals were housed in a controlled environment (22 °C, 12-h light/dark cycle) with free access to water and a standard diet containing 8.8 g/kg calcium and 5.9 g/kg phosphate. The LPS group was intraperitoneally injected with LPS (L8880, Solarbio, China; 5 mg/kg) on days 0 and 4. The electromagnetized AuNP group was intravenously injected with AuNPs (60 μg/kg) on days 1, 3, and 5 following LPS administration and then exposed to a PEMF for 4 h daily, while control and LPS groups were sham-exposed. On day 8, all mice were euthanized, and their right femoral specimens were harvested for micro-computed tomography (CT) analysis, histological evaluation, and immunohistochemical (IHC) staining.

### Micro-CT analysis

Following euthanasia, the right femurs were isolated, fixed in 4% paraformaldehyde for 48 h, and stored in 70% ethanol. High-resolution micro-CT imaging was conducted with SkyScan1276 (Bruker), generating 2D cross-sectional images. Morphometric parameters, analyzed using CTAn software (Bruker), included both 2D and 3D measurements at consistent anatomical locations of the femur across groups. Key metrics analyzed included size, major diameter, perimeter, form factor, orientation, bone mineral density (BMD), percent bone volume (BV/TV), bone surface density (BS/TV), trabecular thickness (Tb. Th), trabecular separation (Tb. Sp), and trabecular number (Tb. N). Additionally, the 3-Matic software (Materialise, Belgium) facilitated 3-dimensional (3D) reconstruction and visualization of trabecular bone regions across experimental groups.

### Cytokine detection

The concentrations of TNF-α, IL-6, and IL-12 in the serum samples were quantified using an enzyme-linked immunosorbent assay (ELISA) kit (Invitrogen) following the manufacturer’s instructions. Briefly, 20 μl of diluted serum was added to enzyme-coated wells, followed by incubation with standard enzyme reagents at 37 °C for 20 min. After 5 washes, the chromogenic substrate was added and incubated for 10 min at 37 °C. The reaction was terminated with a stop solution (the blue color immediately turns to yellow at this time), and absorbance at 450 nm was measured within 15 min using a microplate reader (SpectraMax i3x, Molecular Devices). Cytokine concentrations were calculated based on a standard curve.

### Histological and IHC staining

The fixed femurs were decalcified in 10% EDTA solution at room temperature for 2 weeks until the bone tissue was fully softened, then rinsed, dehydrated, cleared, embedded in paraffin, and sectioned to 5-μm thickness. The sections subsequently underwent hematoxylin and eosin (H&E) staining, Masson’s trichrome staining, and IHC staining. For H&E staining, the sections were deparaffinized, rehydrated, stained with hematoxylin for 10 min, differentiated, stained with eosin, dehydrated, cleared, and mounted. Masson’s trichrome staining followed similar initial steps, with sequential staining using hematoxylin, Biebrich scarlet-acid fuchsin, and aniline blue, finalized by dehydration, clearing, and mounting. In IHC staining, sections were deparaffinized, rehydrated, subjected to microwave antigen retrieval in citrate buffer, and treated with 3% hydrogen peroxide to quench endogenous peroxidase activity. Blocking was performed with 5% goat serum-minimized nonspecific binding. Subsequently, the sections were incubated overnight at 4 °C with diluted primary antibodies targeting RUNX2 (1:200), COL1 (1:200), and OPN (1:300). After the sections were washed, they were incubated with HRP-conjugated secondary antibodies, developed with DAB (3,3′-diaminobenzidine), and counterstained with hematoxylin, dehydrated, cleared, and mounted. The stained sections were imaged under an upright light microscope (DM6000B, Leica).

### Statistical analysis

All data were expressed as the mean ± SD with sample sizes indicated in the figures and/or their legends. Statistical analyses were performed using GraphPad Prism 9.5.0. For 2-group comparisons, statistical analyses were performed using the Student’s *t* test. One-way and 2-way analysis of variance (ANOVA) was performed to compare the effects of more than 2 groups. Significance levels were indicated as *P < 0.05, **P < 0.01, and ***P < 0.001.

## Results

### Synthesis, characterization, and biocompatibility of electromagnetized AuNPs

**Fig. 2.**
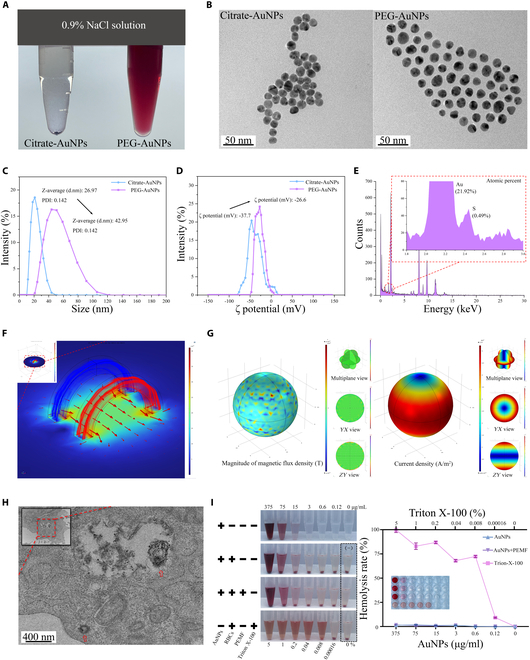
The characterization and biocompatibility of electromagnetized AuNPs. (A) Due to the replacement of surface citrates by the PEG groups, AuNPs acquired enhanced stability in physiological conditions. (B) Representative TEM image of citrate-AuNPs and PEG-AuNPs. (C and D) The changes in hydrodynamic diameter and ζ potential after functionalization were measured using DLS. (E) The successful conjugation of the PEG groups onto the nanoparticles was confirmed by EDS. (F) Calculation of the magnetic flux generated by the EMF system with Helmholtz coils. The red arrows are magnetic field vectors that represent the direction and intensity of the generated EMF. (G) Calculation of the magnitude and spatial distribution of the magnetic flux density and current density on the surface of AuNPs during EMF exposure. (H) Representative bio-TEM image of MC3T3-E1 cells treated with electromagnetized AuNPs. The red arrows indicate that the AuNPs were taken up via endocytosis and distributed within intracellular vesicles, with some escaping into the cytoplasm. (I) RBC hemolysis assay demonstrated the excellent blood compatibility potential of different concentrations of electromagnetized AuNPs.

To enhance stability under physiological environments, we first prepared PEG-AuNPs by replacing citrate with carboxylated PEG using Au–S bonds (Fig. 2A and Fig. S2). The TEM imaging (Fig. 2B) confirmed that the PEG-AuNPs exhibited a spherical morphology and were well-dispersed, with an average diameter of 16.34 ± 2.13 nm (Fig. S3). The modified AuNPs displayed a distinct UV–vis absorption peak at 520 nm, without broadening the half-peak width (Fig. S4), indicating stable dispersion in pure water. The PEG modification improved AuNP dispersibility through steric effects, thereby increasing the hydrodynamic particle size from 26.97 ± 6.31 nm to 42.95 ± 16.34 nm and altering their ζ potential from −37.70 ± 16.80 mV to −26.60 ± 10.00 mV (Fig. 2C and D). The changes in the average hydrodynamic particle size and ζ potential are shown in Fig. S5. Energy-dispersive x-ray spectroscopy (EDS) and XRD analyses validated successful PEG conjugation by detecting sulfur on the AuNP surface (Fig. 2E and Fig. S6). Cytotoxicity tests indicated that AuNPs were nontoxic at concentrations between 0.12 and 75 μg/ml, with 3 μg/ml significantly improving cell viability (Fig. S7). Consequently, a concentration of 3 μg/ml AuNPs was selected for subsequent experiments.

For EMF exposure, an electromagnetic generation system was employed to produce consistent electromagnetic stimulation of varying intensities at the coil’s center (Fig. 2F and Fig. S8). As shown in Fig. S9, the waveform utilized in this study is generated on the basis of the low-frequency single-pulsed EMF that is modulated by another high-frequency carrier wave. We then calculated the magnetic field intensity and current density distribution on the surface of EMF-exposed AuNPs. The results unveiled significant changes in magnetic field intensity and current density, which confirmed the electromagnetic responsiveness of AuNPs to EMF exposure (Fig. 2G and Fig. S10). A peak magnetic flux density of 2 mT during a 2-h PEMF exposure significantly enhanced cell viability and was thus adopted for further studies (Fig. S11).

The electromagnetized AuNPs were internalized by MC3T3-E1 cells through endocytosis, where they were distributed within intracellular vesicles, with some particles entering the cytoplasm (Fig. 2H). Interestingly, PEMF exposure increased the AuNP uptake by 1.23-fold in MC3T3-E1 cells compared to untreated cells (4.82 ± 0.28 ng/10^6^ cells versus 3.93 ± 0.17 ng/10^6^ cells; Fig. S12). In terms of cytocompatibility, electromagnetized AuNPs exhibited no adverse effects on cell viability and survival following 2 h of daily exposure for 3 or 7 d (Figs. S13 and S14). Moreover, the electromagnetized AuNPs demonstrated excellent hemocompatibility (Fig. 2I) and did not induce any notable pathological toxicity in the major organs after 2 weeks of treatment (Fig. S15).

### Electromagnetized AuNPs enhance osteogenesis in vitro

**Fig. 3.**
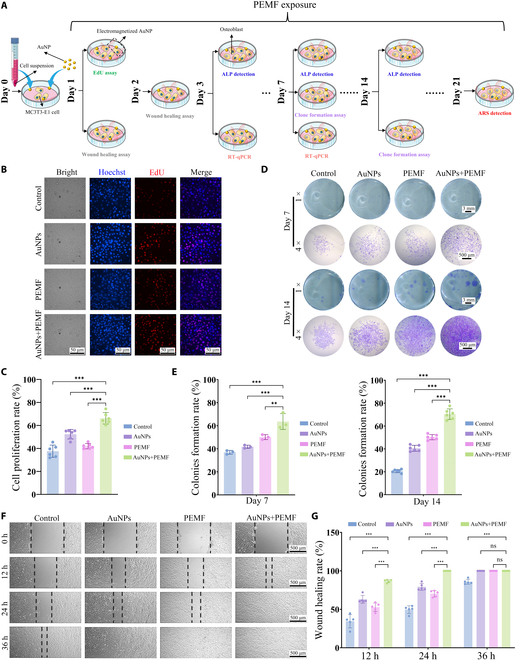
Electromagnetized AuNPs improve cellular biological functions. (A) Schematic diagram of the experimental design. (B) Representative images of EdU assay. The red fluorescent probe is utilized to label newly synthesized DNA, signifying cells that are actively undergoing proliferation. (C) Quantitative analysis of EdU assay. (D) Representative macroscopic images (1×, no magnification) and microscope images (4× objective lens) of the colony formation experiment. (E) Quantitative analysis of colony formation experiment. (F) Representative images of wound healing assay. (G) Quantitative analysis of the wound healing assay. A one-way ANOVA was performed for (C) and (E), while a 2-way ANOVA was conducted for (G). Statistical significance is denoted as **P < 0.01 and ***P < 0.001.

Effective bone formation and repair depend on rapid osteoblast proliferation, migration, and osteogenic differentiation, especially in bone disorders and injuries. The experimental design to investigate the effects of electromagnetized AuNPs on these parameters is shown schematically in Fig. 3A. Our results indicated that the electromagnetized AuNPs significantly enhanced cell proliferation (Fig. 3B and C) and promoted single-cell colony formation (Fig. 3D and E), supporting long-term growth and survival. Furthermore, the electromagnetized AuNPs significantly accelerated cell migration, achieving complete closure of a 1,000-μm wound within 24 h—significantly faster than the control group, which required over 36 h for similar wound closure (Fig. 3F and G). These findings suggest that electromagnetized AuNPs facilitate bone regeneration by boosting cell growth and migration.

**Fig. 4.**
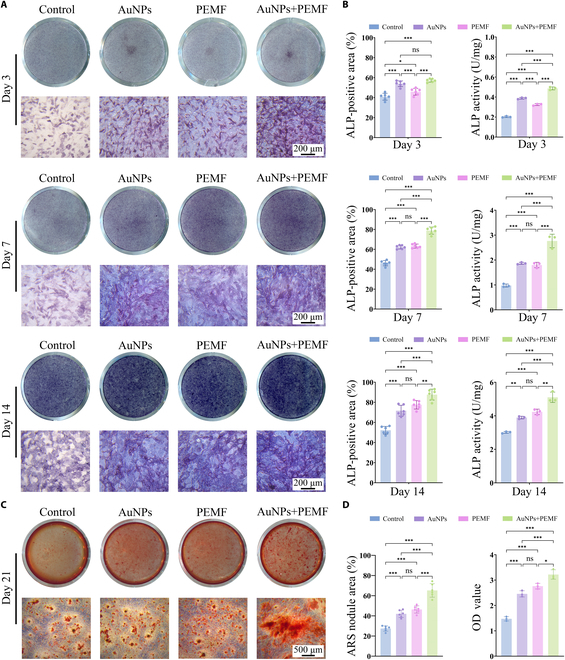
Electromagnetized AuNPs promote osteogenic differentiation. (A) Representative macroscopic images (1×, no magnification) and microscope images (10× objective lens) of the ALP staining after osteogenic induction at 3, 7, and 14 d. (B) Quantification of ALP-positive area and ALP activity assays. (C) Representative macroscopic images (1×, no magnification) and microscope images (4× objective lens) of the ARS staining after osteogenic induction at 21 d. (D) Quantification of ARS nodule area and calcium deposition detection. A one-way ANOVA was conducted, with significance levels indicated as *P < 0.05, **P < 0.01, and ***P < 0.001.

To assess osteogenic differentiation, ALP expression—an indicator of osteoblast maturation, was evaluated through staining and activity assays (Fig. 4A and B). The electromagnetized AuNP group demonstrated the highest ALP-positive area and ALP activity at each time point, indicating that this treatment effectively enhances ALP expression. In detail, by day 3, the ALP-positive area and ALP activity in the AuNP group were significantly higher than those in the PEMF group. By days 7 and 14, a progressive elevation in ALP expression was observed in the PEMF group, reaching levels comparable to or potentially exceeding those in the AuNP group, suggesting a cumulative effect of PEMF on osteogenic differentiation with extended exposure. Notably, the electromagnetized AuNP group exhibited a significant advantage in early osteogenic differentiation, with ALP expression markedly higher than that of either AuNPs or PEMF alone, potentially compensating for the delayed onset of PEMF effects. As the culture period progressed, ALP levels in the electromagnetized AuNP group continued to rise and consistently remained higher than those in the single-treatment groups, further indicating the synergistic effect of AuNPs and PEMF in promoting osteogenic differentiation.

ARS staining was performed to evaluate late-stage mineralization by visualizing calcium deposition, which appeared as bright orange-red mineralized nodules. By day 21, MC3T3-E1 cells treated with electromagnetized AuNPs exhibited the highest mineralization capacity, with significantly more and larger mineralized nodules (Fig. 4C). Quantitative analysis further confirmed that the ARS-stained area and calcium salt formation in this group were markedly higher than those in the other groups (Fig. 4D), underscoring the combined treatment’s effectiveness in synergistically enhancing differentiation and mineralization.

### Molecular mechanisms of osteogenesis enhanced by electromagnetized AuNPs

**Fig. 5.**
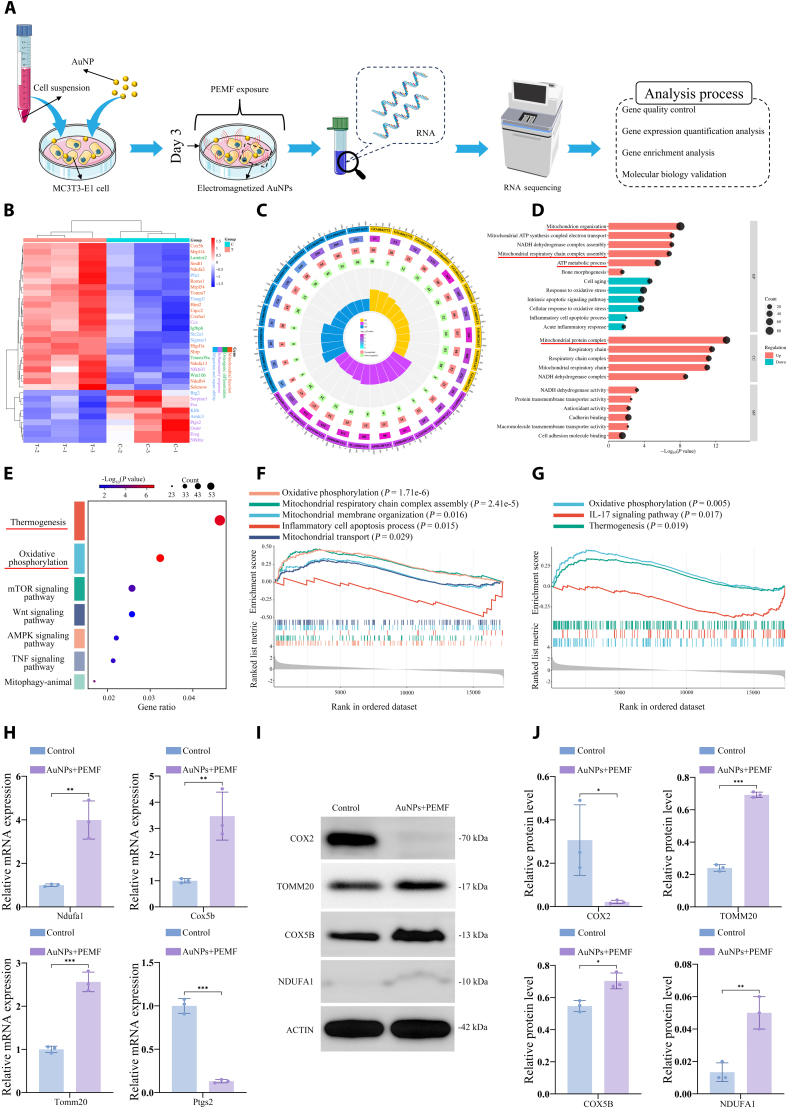
Transcriptomic and molecular validation of osteogenesis induced by electromagnetized AuNPs. (A) Schematic representation of the experimental steps and analysis process. (B) Cluster heatmap of the top 100 significant DEGs (after removing unannotated or functionally unclear genes). The color blocks represent the fold change of gene expression, with red indicating up-regulation and blue indicating down-regulation. The deeper the color, the greater the fold change. (C) Enrichment circle plot of the GO analysis. The outer ring highlights the top 10 most significant GO terms within the categories of BP, CC, and MF, depicted in yellow, purple, and blue, respectively. The second ring represents the number of genes in the genomic background and the associated *P* values for gene enrichment for each GO term. The third and fourth rings display the counts of up-regulated and down-regulated genes corresponding to each GO term, respectively. The inner ring presents a bar graph illustrating the enrichment factor for each GO term. The category and detailed description of each GO term are provided in Table S2. (D) Bar bubble plot of GO functional enrichment. The length of each bar represents the significance of enrichment, with red bars indicating up-regulated terms and blue bars indicating down-regulated terms. The size of the black bubbles corresponds to the number of enriched genes. (E) The bubble plot illustrates the enriched pathways of DEGs after electromagnetized AuNP treatment. Bubble size represents the number of genes associated with each pathway, while bubble color indicates significance of enrichment. (F) GSEA of GO-BP reveals a significant up-regulation of mitochondrial function gene sets after treatment with electromagnetized AuNPs. (G) GSEA of KEGG shows a significant up-regulation of OXPHOS and thermogenesis pathways, with the down-regulation of the IL-17 signaling pathway following the electromagnetized AuNP treatment. (H) RT-qPCR and (I) WB analysis of NDUFA1, COX5B, TOMM20, and COX2 expression following electromagnetized AuNP treatment for 3 d. (J) Quantification of WB analysis. A 2-sample *t* test was performed, with significance levels indicated as *P < 0.05, **P < 0.01, and ***P < 0.001.

To explore the molecular mechanisms by which electromagnetized AuNPs enhance cellular biological functions and promote osteogenic differentiation, RNA-sequencing analysis was conducted (Fig. 5A). This analysis identified 1,662 up-regulated and 1,739 down-regulated DEGs following treatment with electromagnetized AuNPs (Fig. S17). Cluster analysis of the significant DEGs was performed to identify expression patterns and biological relevance. The results indicated that these DEGs are primarily associated with mitochondrial function, osteogenic differentiation, inflammation, migration, and cellular repair (Fig. 5B).

To further elucidate the biological functional changes induced by differential gene expression, we performed GO functional enrichment analysis on these DEGs. As shown in Fig. 5C and Table S2, GO enrichment analysis revealed that the most significantly enriched terms were primarily linked to ATP synthesis-coupled electron transport in biological processes (BP), mitochondrial respiratory chain complex in cellular components (CCs), and NADH dehydrogenase activity in molecular functions (MFs). The trend analysis indicated that the up-regulated DEGs, in response to electromagnetized AuNP treatment, were predominantly enriched in mitochondrial-related processes and components, including mitochondrial biogenesis, respiratory chain complex assembly, ATP metabolism, and mitochondrial protein complexes (Fig. 5D). In contrast, the DEGs associated with oxidative stress response, apoptosis, and inflammation were down-regulated, implying a reduction in cellular stress and inflammatory responses. KEGG pathway enrichment analysis showed that DEGs following electromagnetized AuNP treatment were significantly enriched in multiple pathways beneficial for osteogenesis, such as OXPHOS, thermogenesis, and the Wnt signaling pathway, compared with the untreated group (Fig. 5E).

Additionally, GSEA for BP and MF revealed that electromagnetized AuNPs could significantly up-regulate gene sets related to OXPHOS, mitochondrial organization, and the respirasome while down-regulating inflammatory and apoptotic processes (Fig. 5F and Fig. S17). Moreover, GSEA results for MF indicated that electromagnetized AuNPs modulated the extracellular matrix (ECM) structure and transmembrane transport functions (Fig. S18). Beyond the significant up-regulation of mitochondrial metabolic pathways, GSEA based on KEGG further demonstrated that the IL-17 signaling pathway was notably enriched and down-regulated (Fig. 5G).

To validate the bioinformatics findings, quantitative reverse transcription PCR (RT-qPCR) and Western blotting (WB) analyses were conducted to assess the expression of mitochondrial proteins involved in OXPHOS and the crucial factor in the IL-17 signaling pathway. The results confirmed that both mRNA and protein levels of NDUFA1, COX5B, and TOMM20 were significantly elevated following electromagnetized AuNP treatment compared to the controls (Fig. 5H and I). In parallel, RT-qPCR and WB analyses demonstrated that Ptgs2, which encodes COX2 and functions as a key downstream factor in the IL-17 signaling pathway, was significantly down-regulated after treatment with electromagnetized AuNPs. These findings suggest that electromagnetized AuNPs enhance osteoblast biological function and differentiation potential by promoting mitochondrial OXPHOS while alleviating inflammation through inhibition of the IL-17 signaling pathway, which may help create a more favorable osteogenic environment.

### Electromagnetized AuNPs mitigate inflammation-induced osteogenic impairment by restoring mitochondrial function

**Fig. 6.**
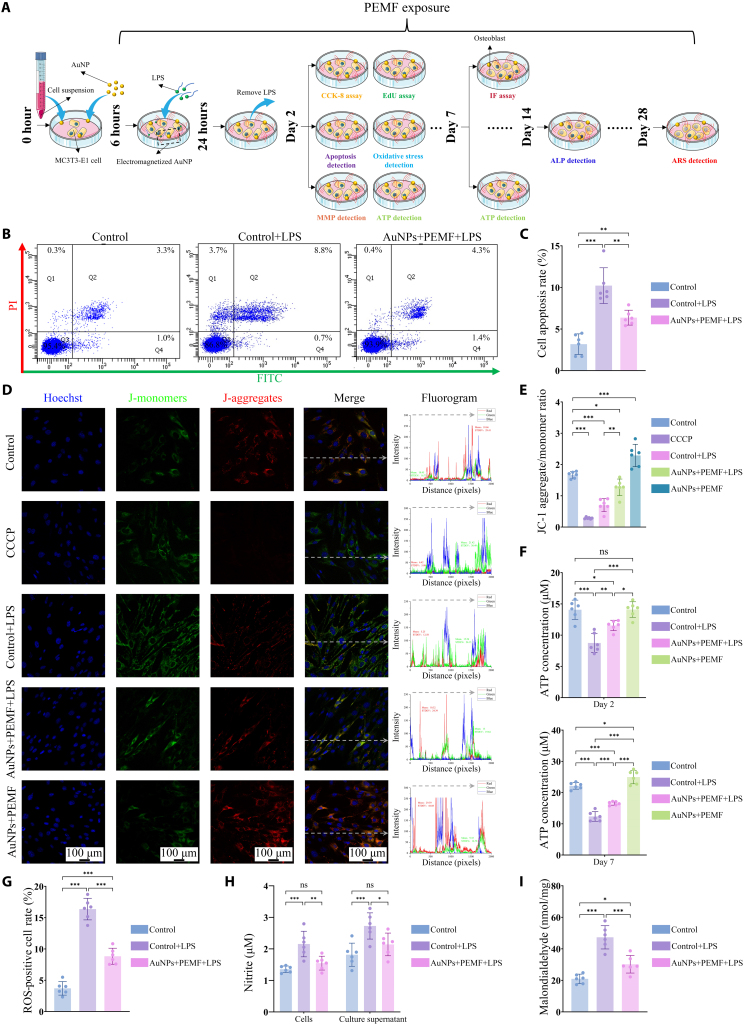
Electromagnetized AuNPs restore mitochondrial homeostasis disrupted by inflammatory stimuli. (A) Schematic diagram of the experimental design. (B and C) Flow cytometry was employed to assess cell apoptosis under various treatments using the Annexin V-FITC assay. (D) Representative images and fluorescence intensity analysis of JC-1 staining. Red fluorescence indicates J-aggregates, while green fluorescence indicates J-monomers. (E) JC-1 aggregate/monomer ratio in different treatment groups. (F) Measurement of ATP content in different treatment groups. (G) Quantitative analysis of the ROS levels, as measured using the DCFH-DA fluorescent probe method. (H) Intracellular and culture supernatant NO levels were quantified using the classical Griess reagent method. (I) MDA levels in cell lysates were quantified using colorimetric analysis. A one-way ANOVA was performed for (C), (E), (F), (G), and (I), while a 2-way ANOVA was conducted for (H). Statistical significance is denoted as *P < 0.05, **P < 0.01, and ***P < 0.001.

Inflammatory responses are critical contributors to OP progression, as they impair the biological function and differentiation potential of osteoblasts, leading to decreased bone density. Inflammation not only suppresses cell survival and osteogenic differentiation but also disrupts mitochondrial homeostasis, further exacerbating bone loss. Therefore, mitigating inflammation’s detrimental effects on osteogenesis is essential for OP prevention and treatment. To this end, we established an in vitro inflammatory microenvironment using LPS stimulation and subsequently evaluated the effects of electromagnetized AuNPs on cell proliferation, apoptosis, mitochondrial function, oxidative stress, and osteogenic differentiation (Fig. 6A).

Cell viability and proliferation were assessed by using CCK-8 and EdU assays. These results demonstrated that LPS treatment significantly inhibited cell vitality and proliferation, while electromagnetized AuNPs effectively counteracted these negative effects (Figs. S19 to S21). Similarly, the Ki67 protein expression mirrored these trends (Figs. S22 and S23), thereby confirming that electromagnetized AuNPs promote cell proliferation even under LPS-induced inflammatory stress. Moreover, electromagnetized AuNPs markedly attenuated LPS-induced cell apoptosis, lowering the rate from 10.22 ± 2.14% to 6.37 ± 0.89% (Fig. 6B and C). These results suggest that electromagnetized AuNPs exert a cytoprotective effect in an inflammatory microenvironment.

Considering the potential of electromagnetized AuNPs to improve mitochondrial function, we next evaluated MMP using the JC-1 fluorescent probe to determine whether electromagnetized AuNPs could mitigate LPS-induced mitochondrial damage. As shown in Fig. 6D, LPS stimulation, similar to CCCP treatment, led to a noticeable increase in green JC-1 monomers when compared with the control, indicating MMP depolarization. However, electromagnetized AuNPs reversed these changes and raised the JC-1 aggregate/monomer ratio from 0.70 ± 0.21 to 1.27 ± 0.26, suggesting restoration of MMP (Fig. 6E). We subsequently assessed the effects of electromagnetized AuNPs on the ultimate bioenergetic effect during the proliferative and differentiation phases. As illustrated in Fig. 6F, treatment with electromagnetized AuNPs markedly restored ATP production suppressed by LPS stimulation during both phases. We further assessed oxidative stress markers by measuring the ROS, NO, and MDA levels. LPS treatment induced excessive ROS, NO, and MDA production, while electromagnetized AuNPs restored these levels to baseline values (Fig. S24 and Fig. 6G to I). These findings indicate that, under inflammatory conditions, electromagnetized AuNPs effectively stabilize MMP, reduce oxidative stress, and enhance cellular energy production during osteogenic differentiation. To explore the effects of electromagnetized AuNPs in more detail, we analyzed the MMP and ATP levels without LPS-induced inflammation. The results demonstrated that the electromagnetized AuNP group exhibited both the highest JC-1 aggregate/monomer ratio (2.29 ± 0.36 versus 1.67 ± 0.11) and markedly elevated ATP levels on day 7 compared to control (24.97 ± 2.16 μM versus 22.14 ± 1.04 μM). These results are consistent with the transcriptomic analysis, further confirming that electromagnetized AuNPs enhance mitochondrial function, thereby improving energy metabolism, which may act as a driving force for accelerated osteogenic differentiation.

**Fig. 7.**
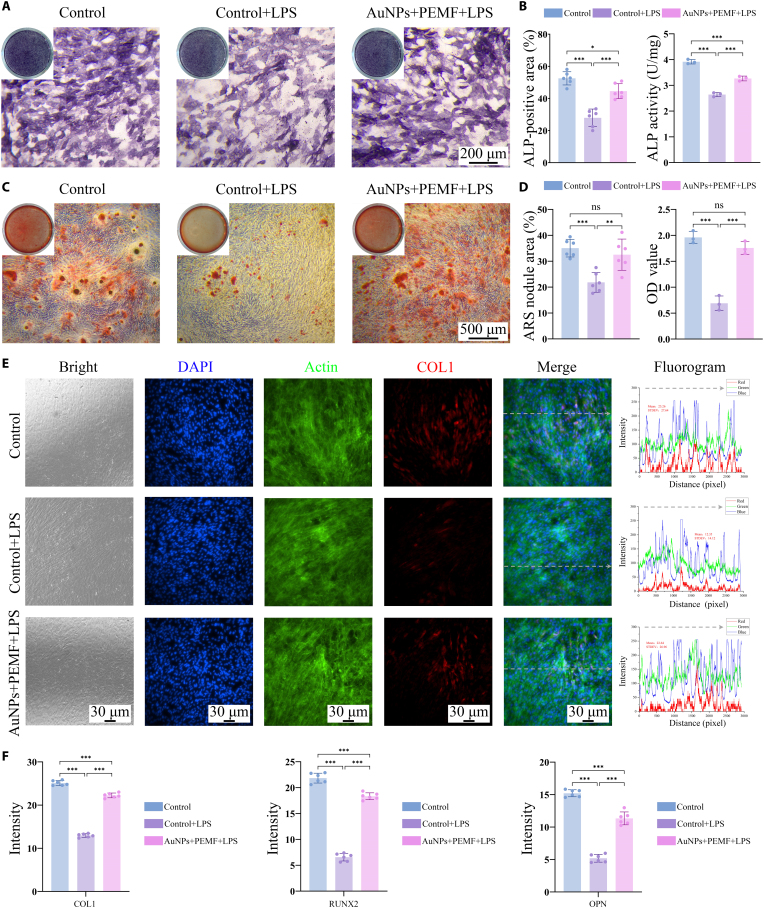
Electromagnetized AuNPs boost osteogenic differentiation under the inflammatory environment. (A and B) ALP staining, activity, and quantification analysis after 14 d of osteogenic induction under inflammatory conditions. (C and D) ARS staining, calcium deposition, and quantification analysis after 21 d of osteogenic induction under inflammatory conditions. (E) Representative images and fluorescence intensity analysis of COL1 protein IF staining. (F) Quantitative analysis of COL1, RUNX2, and OPN protein IF staining. A one-way ANOVA was performed, with significance levels indicated as *P < 0.05, **P < 0.01, and ***P < 0.001.

The osteogenic potential of electromagnetized AuNPs under inflammatory conditions was subsequently investigated. By day 14 (Fig. 7A), the ALP-positive staining was markedly reduced in the LPS group, indicating that the inflammatory microenvironment significantly suppressed ALP expression. Electromagnetized AuNP treatment effectively reversed this suppression, increasing the ALP-positive area from 27.93 ± 5.46% to 44.55 ± 4.63% and ALP activity from 2.64 ± 0.08 U/mg to 3.27 ± 0.09 U/mg (Fig. 7B). By day 28, electromagnetized AuNPs effectively mitigated the inhibitory effects of LPS on osteogenic differentiation and markedly enhanced ECM mineralization (Fig. 7C). The ARS-positive staining area increased from 21.85 ± 3.81% to 32.53 ± 6.02%, and calcium deposition exhibited a 1.55-fold increase compared to the LPS group (1.76 ± 0.12 versus 0.69 ± 0.14). Notably, there was no statistically significant difference in calcium deposition between the electromagnetized AuNP-treated group and the control group (Fig. 7D), indicating that the treatment nearly restored mineralization capacity to physiological levels. Furthermore, IF staining revealed that electromagnetized AuNPs alleviated the LPS-induced down-regulation of COL1, RUNX2, and OPN to 83.97%, 88.57%, and 74.56% of control levels, respectively (Fig. 7E and F and Figs. S25 and S26). Taken together, electromagnetized AuNPs exhibited a notable capacity to counteract LPS-induced suppression of osteogenic differentiation by promoting ALP expression, enhancing matrix mineralization, and restoring the expression of key osteogenic markers. These findings suggest that this approach maintains osteogenic potential of osteoblasts even under inflammatory conditions.

### Electromagnetized AuNPs improve bone mass loss and microstructural deterioration in an OP mouse model

**Fig. 8.**
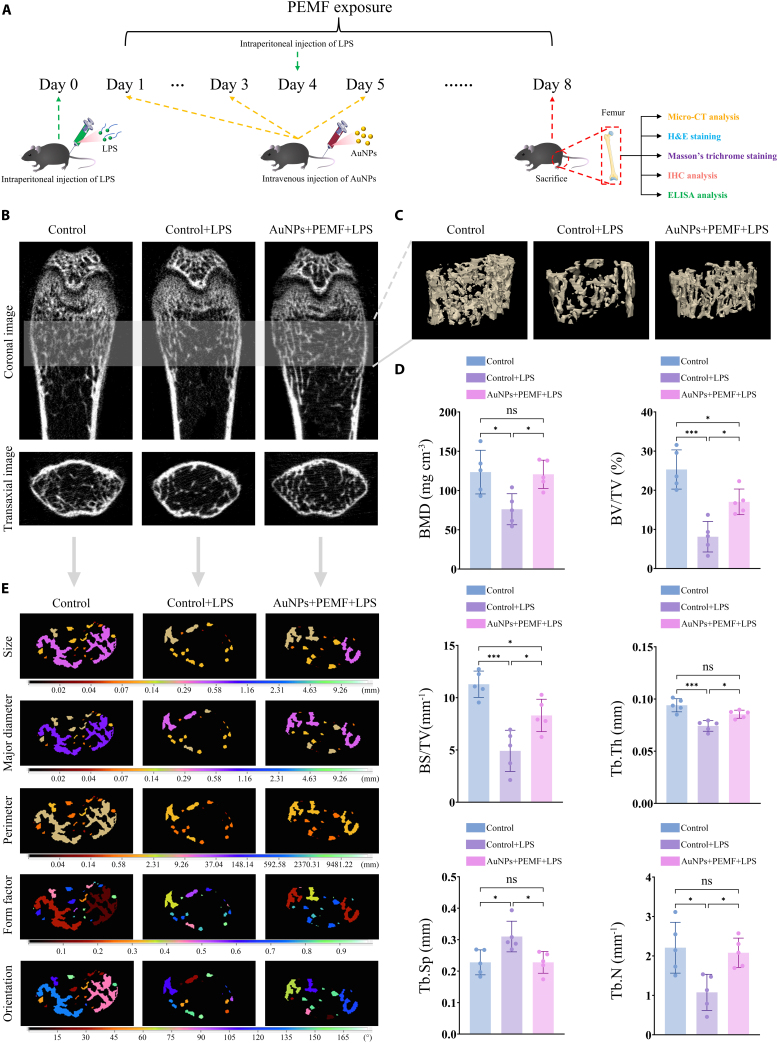
Electromagnetized AuNPs mitigate bone mass loss and trabecular structural deterioration in an OP mouse model. (A) Schematic representation of experimental procedure and timeline. (B and C) Representative micro-CT and 3D reconstruction images of each group. (D) Quantitative analysis of the representative morphometric parameters of bone trabeculae, as measured by micro-CT, to evaluate the therapeutic impact of electromagnetized AuNPs. (E) Visualization of the distribution of 2D morphometric parameters in bone trabeculae. The parameters include size, major diameter, perimeter, form factor, and orientation. Colored regions represent trabecular segments, with parameter values presented as heatmaps to visualize intergroup differences. A one-way ANOVA was performed, with significance levels indicated as *P < 0.05 and **P < 0.01.

Before evaluating the in vivo therapeutic effects, we first examined whether intravenously administered AuNPs could effectively reach bone tissue. ICP-MS analysis showed that, at the treatment dose of 60 μg/kg, Au content in bilateral hindlimb bones reached 63.37 ± 4.65 ng/g at 6 h post-injection and remained detectable at 29.41 ± 3.15 ng/g at 24 h (*P* < 0.05). These results confirm that AuNPs are capable of accumulating in bone tissue following systemic administration, thereby providing a basis for their potential in vivo therapeutic action. To further evaluate the in vivo therapeutic potential of electromagnetized AuNPs, we examined their effects in an LPS-induced OP mouse model. The experimental procedure and timeline are shown in Fig. 8A. The micro-CT was employed to assess changes in bone mass and microarchitecture. LPS treatment resulted in significant bone loss, as evidenced by reduced trabecular volume and connectivity, as well as a more dispersed and irregular arrangement (Fig. 8B). In contrast, treatment with electromagnetized AuNPs led to a marked recovery of the trabecular structure, suggesting a protective and regenerative effect, as confirmed by the 3D reconstruction of femur samples (Fig. 8C).

Quantitative analysis of 3D microstructural parameters revealed that LPS significantly impaired trabecular bone architecture, whereas electromagnetized AuNPs effectively ameliorated these structural deficits (Fig. 8D). Specifically, the mean BMD in the LPS group was markedly reduced from 123.40 ± 27.83 mg cm^−3^ in the control group to 76.14 ± 19.78 mg cm^−3^. Treatment with electromagnetized AuNPs restored BMD to 120.60 ± 17.98 mg cm^−3^, representing a 58.39% increase relative to the LPS group and approaching baseline levels observed in controls. In addition, LPS resulted in a substantial decrease in BV/TV and BS/TV, which declined to 32.13% and 43.49% of control values, respectively (BV/TV: 25.30 ± 5.00% versus 8.13 ± 3.90%; BS/TV: 11.29 ± 1.26 mm^−1^ versus 4.91 ± 1.97 mm^−1^). Following treatment, BV/TV and BS/TV were significantly improved, recovering to 67.35% and 73.52% of control values, respectively (BV/TV: 17.04 ± 3.27%; BS/TV: 8.30 ± 1.55 mm^−1^). Moreover, LPS stimulation disrupted trabecular microarchitecture by reducing Tb. Th from 94.00 ± 6.28 μm to 74.21 ± 5.08 μm, increasing Tb. Sp from 0.23 ± 0.04 mm to 0.31 ± 0.05 mm, and decreasing Tb. N from 2.21 ± 0.64 mm^−1^ to 1.08 ± 0.46 mm^−1^. The electromagnetized AuNPs effectively reversed these alterations, with Tb. Th increasing to 85.43 ± 3.88 μm, Tb. Sp decreasing to 0.23 ± 0.03 mm, and Tb. N elevating to 2.08 mm^−1^.

2D morphometric analysis of femoral cross-sections further confirmed these findings (Fig. 8E). In the control group, the size of trabeculae was mainly 0.29 mm, with a major diameter of 0.58 mm and a perimeter of 2.31 mm. LPS-induced inflammation markedly reduced trabeculae size, major diameter, and perimeter to 0.02 to 0.14 mm, 0.02 to 0.29 mm, and 0.04 to 0.58 mm, respectively. In contrast, the electromagnetized AuNP effectively restored these parameters to 0.14 to 0.29 mm, 0.29 to 0.58 mm, and 0.58 to 2.31 mm, respectively. Moreover, trabeculae in the LPS group appeared fragmented and more elliptical in shape, with form factor approaching 1, and exhibited a scattered distribution with disorganized orientation. Following treatment with electromagnetized AuNPs, the structural integrity of trabeculae was markedly improved, with the form factor trending toward 0 and the alignment becoming more uniform, indicating a substantial recovery of trabecular microarchitecture.

**Fig. 9.**
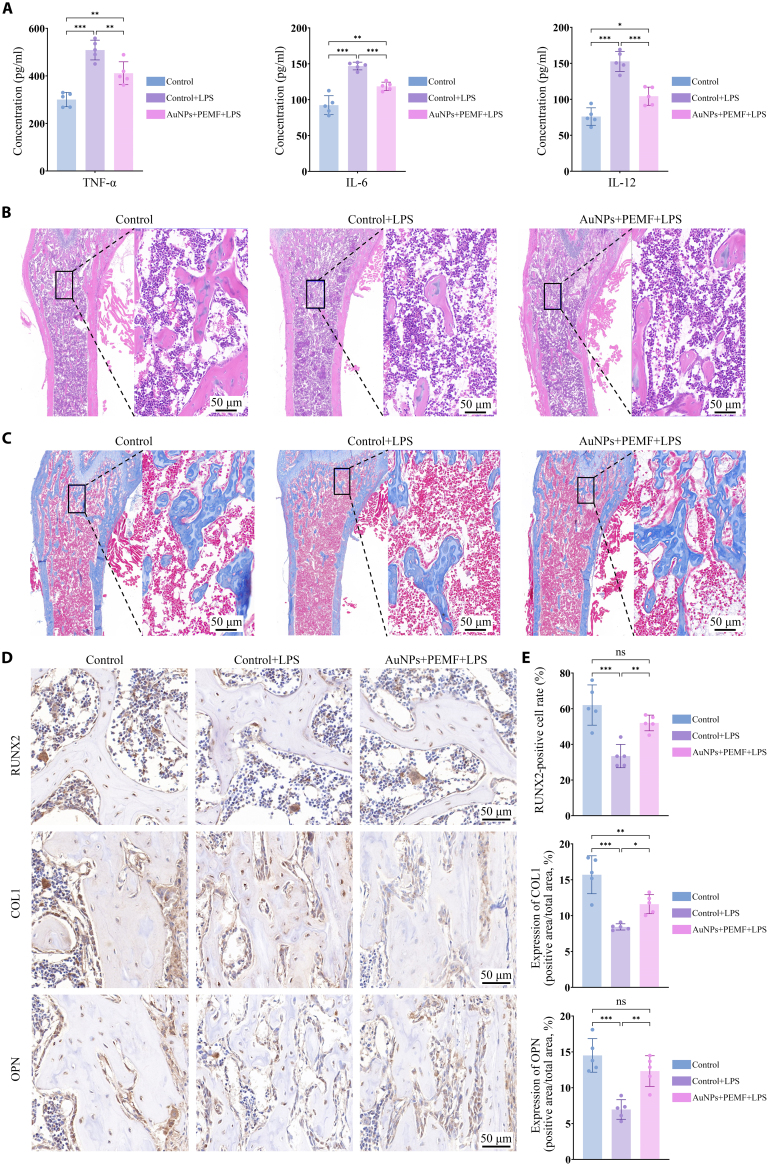
Electromagnetized AuNPs alleviate inflammation and promote bone regeneration in an OP mouse model. (A) ELISA analysis of serum levels of TNF-α, IL-6, and IL-12. (B) Representative H&E staining images of femoral sections from each group. (C) Representative Masson’s trichrome-stained images of femoral sections. (D) Representative IHC staining images of osteogenic markers RUNX2, COL1, and OPN, with brown indicating positive staining. (E) Quantification analysis of RUNX2-positive cells and COL1- and OPN-positive area based on IHC images. A one-way ANOVA was performed, with significance levels indicated as *P < 0.05, **P < 0.01, and ***P < 0.001.

ELISA assays, histological analysis, and IHC analysis were performed to assess the therapeutic efficacy and investigate the underlying molecular mechanisms of electromagnetized AuNPs. As shown in Fig. 9A, TNF-α, IL-6, and IL-12 levels in serum were elevated following LPS stimulation, whereas treatment with electromagnetized AuNPs attenuated the aberrant expression of these pro-inflammatory cytokines. H&E staining revealed that the control group exhibited dense, well-organized trabeculae, while LPS-induced inflammation led to significant trabeculae loss. Electromagnetized AuNPs alleviated this structural disruption and trabeculae loss (Fig. 9B). In parallel, Masson’s trichrome-stained images showed that the trabeculae became more compact, regularly arranged, and uniformly thick, with a well-distributed collagen fiber network following treatment with electromagnetized AuNPs (Fig. 9C). As illustrated in Fig. 9D and E, IHC analysis showed that treatment with electromagnetized AuNPs increased the expression of RUNX2, COL1, and OPN by 55.60%, 37.60%, and 77.01%, respectively, compared to the LPS group. In parallel, to further verify the involvement of IL-17 signaling in the therapeutic effect, RT-qPCR and WB analyses were performed on femoral bone tissue to assess the expression of IL-17A, IL-17F, and IL-17RA. Electromagnetized AuNPs significantly suppressed the LPS-induced up-regulation of all 3 targets at both mRNA and protein levels (Figs. S27 to S29). These results demonstrated the highly efficient antiosteoporosis ability of electromagnetized AuNPs in an LPS-induced OP model, as evidenced by improved bone microarchitecture and enhanced expression of osteogenic proteins.

To further dissect the respective contributions of AuNPs and PEMF, as well as their potential synergy in vivo, we conducted additional animal experiments by including the AuNP + LPS and PEMF + LPS groups. Micro-CT analysis was performed to quantitatively assess bone mass and microarchitecture across all groups. The results showed that both AuNP + LPS and PEMF + LPS treatments improved bone microarchitecture compared to the LPS model group (Fig. S30). Specifically, BMD increased from 65.75 ± 8.67 mg cm^−3^ in the LPS group to 102.4 ± 5.02 mg cm^−3^ and 95.41 ± 4.66 mg cm^−3^, while BV/TV increased from 6.26 ± 1.31% to 13.43 ± 1.50% and 12.23 ± 1.36%, respectively (Fig. S31). Notably, the electromagnetized AuNP-treated group still exhibited the most pronounced therapeutic effect, with bone mass nearly restored to baseline levels. At day 8, the AuNP + LPS group showed slightly higher BMD and BV/TV than did the PEMF + LPS group, although the difference was not statistically significant. These findings support a synergistic interaction between AuNPs and PEMF, further underscoring the enhanced therapeutic potential of electromagnetized AuNPs under inflammatory osteoporotic conditions.

## Discussion

EMF, recognized as a noninvasive clinical treatment modality, has been shown to accelerate bone formation and is approved by the U.S. Food and Drug Administration (FDA) for specific therapeutic uses [41]. Our previous study demonstrated that AuNPs are effective in reducing inflammation [37,39] and inhibiting bone resorption [38]. Prior studies have also demonstrated that AuNPs, as conductive and EMF-active nanomaterials, can effectively enhance cellular reprogramming under specific electromagnetic conditions [42]. For instance, Yoo et al. [35] reported that electromagnetized AuNPs significantly improved the efficiency of dopaminergic neuron induction through their interactions with cell membranes and intracellular pathways. Likewise, these nanoparticles were shown to promote neurogenesis and cognitive function in aged and progeroid mouse brains by increasing the numbers of neural stem and progenitor cells [36]. However, these studies have primarily focused on neurological disorders, and the combined use of EMF and AuNPs has not yet been systematically explored in the context of bone diseases. Building upon these findings, we sought to explore whether combining EMF with AuNPs could synergistically enhance osteogenesis while simultaneously optimizing the bone microenvironment in OP. Our results clearly exhibited that electromagnetized AuNPs could significantly improve the cellular biological functions and osteogenic differentiation in vitro. Unlike conventional EMF therapy, which often suffers from local energy attenuation and inefficient energy concentration at the cellular level, AuNPs can serve as intracellular mediators to facilitate more direct and efficient EMF energy delivery to target cells. Moreover, owing to their biological inertness, internalized AuNPs remain stable within cells for extended periods, thus prolonging the bioavailability and enhancing the long-term modulatory effect of EMF on osteogenesis.

While many other methods have been proposed to safely and effectively promote osteogenesis, we propose that electromagnetized AuNPs offer distinct advantages, such as low toxicity, facile synthesis, high controllability, and the ability to improve the bone microenvironment. According to our results, the electromagnetized AuNPs up-regulated genes associated with mitochondrial OXPHOS, thereby enhancing cellular energy metabolism while concurrently suppressing IL-17-mediated pro-inflammatory signaling. This dual regulatory effect was also reflected in the divergent expression patterns of COX family members: COX2, a pro-inflammatory enzyme and downstream effector of IL-17 signaling, was significantly down-regulated, whereas COX5B, a subunit of mitochondrial cytochrome c oxidase involved in OXPHOS, was up-regulated. These changes suggest that electromagnetized AuNPs simultaneously inhibit inflammatory cascades and enhance mitochondrial bioenergetics, further supporting their potential in restoring the bone microenvironment. Consistent with these findings, prior work reported that alkyl-terminated AuNPs (Au_3_@PEG-octadecyl_30%_ NPs, sub-15 nm) can down-regulate genes that are enriched in the downstream of the IL-17 signaling pathway, leading to reduced inflammation and prevention of psoriasis progression with efficacy comparable to standard steroid or vitamin D-based therapies [43]. These data collectively suggest that AuNPs may exert anti-inflammatory effects and broadly modulate inflammatory responses across diverse pathological conditions by targeting the IL-17 signaling pathway. It is also worth noting that AuNPs exhibited stronger effects in short-term experiments (e.g., cell proliferation, migration, and ALP activity at day 3), while EMF showed greater influence in longer-term assays (e.g., matrix mineralization and ALP activity after day 7). This difference may stem from their distinct biological dynamics. Once internalized by cells, AuNPs can rapidly reduce ROS levels and alleviate oxidative stress [44], thereby facilitating early-stage cellular recovery and osteogenic activation. In contrast, the biological effects of EMF depend on sustained stimulation and gradually modulate intracellular signaling pathways [20], which are more directly associated with the progressive induction of osteogenic differentiation and subsequent matrix formation. Moreover, the early cellular improvements triggered by AuNPs may establish a favorable foundation for subsequent EMF exposure, which then helps sustain and amplify osteogenic processes over time.

To further assess whether these nanoparticles could mitigate inflammation-induced osteogenic impairment, LPS was used to simulate an inflammatory environment in vitro. The electromagnetized AuNPs alleviated the detrimental effects of LPS, including reduced cell proliferation, enhanced apoptosis, impaired mitochondrial function, and suppressed osteogenic capacity. Interestingly, compared with the control group, the AuNP + PEMF + LPS group showed increased cell viability in the CCK-8 assay, while flow cytometry revealed elevated levels of apoptosis. These seemingly contradictory results likely arise from the dominant contribution of enhanced metabolic activity in nonapoptotic subpopulations under the influence of electromagnetized AuNPs, leading to an overall increase in CCK-8 signal. Taken together, these results suggest that electromagnetized AuNPs may represent a promising therapeutic strategy for promoting bone formation in inflammatory contexts, thereby mitigating bone loss in OP.

Encouraged by these in vitro findings, we extended our investigation to an in vivo mouse model of LPS-induced OP. This classical model [45,46], characterized by acute inflammation and rapid bone loss, was selected to establish an in vivo inflammatory context. In this setting, AuNPs may exert immediate regulatory effects, whereas PEMF, whose therapeutic activity is known to be time-dependent, may require sustained exposure for full efficacy [47–49]. Notably, AuNPs may also facilitate the localized transmission of electromagnetic energy, thereby improving cellular responsiveness to PEMF. This synergistic interaction may partly explain the superior bone-preserving capacity of electromagnetized AuNPs, which effectively reversed bone mass loss and restored trabecular structural integrity following inflammatory damage. ELISA results confirmed a significant reduction in proinflammatory cytokine secretion, while histological and IHC analyses demonstrated marked improvements in bone matrix quality and osteogenic marker expression after treatment with the electromagnetized AuNPs. Notably, type I collagen (COL1), a key structural component of the ECM, was markedly up-regulated in the osteoporotic bone following treatment with the electromagnetized AuNPs. As COL1 expression is known to vary with pathological context and ECM remodeling, this increase suggests matrix regeneration and restoration of bone homeostasis. Interestingly, previous studies have reported contrasting outcomes in other disease models. For example, in a murine model of renal fibrosis, smaller AuNPs (Au_3_-PEG_500_-FA_32_ NPs, 9.6 nm) significantly down-regulated COL1 expression, thereby mitigating pathological tissue fibrosis [50]. These differential effects may be attributed to variations in particle size, surface modifications, and the distinct microenvironmental conditions present in osteoporotic bone versus fibrotic kidney tissue. From a clinical perspective, the electromagnetized AuNPs present a viable therapeutic strategy for OP. This strategy may offer a controllable method to enhance local bone formation, which may be particularly beneficial in anatomically vulnerable regions prone to osteoporotic fractures, such as the wrist, hip, and thoracolumbar spine. In summary, our findings highlight the synergistic effects of electromagnetized AuNPs—namely, promoting osteogenic differentiation and optimizing the bone microenvironment—as a novel and effective therapeutic approach for OP.

## Ethical Approval

This study was approved by the Animal Ethics Committee of the Capital Medical University (no. AEEI-2023-283). All animals were kept, and experiments were conducted in strict accordance with the regulations.

## Data Availability

The data that support the findings of this study are available from the corresponding author upon reasonable request.
