# Supplementary material for: Enhancing Osteogenesis in Osteoporosis via Electromagnetized Gold Nanoparticles
Source: Biomater Res. 2025 Sep 24;29:0260. doi: 10.34133/bmr.0260 (PMC12459909; doi:10.34133/bmr.0260)
Supplement: Supplementary 1 — Figs. S1 to S31 Tables S1 and S2 [file bmr.0260.f1.docx]

**Supporting Information**


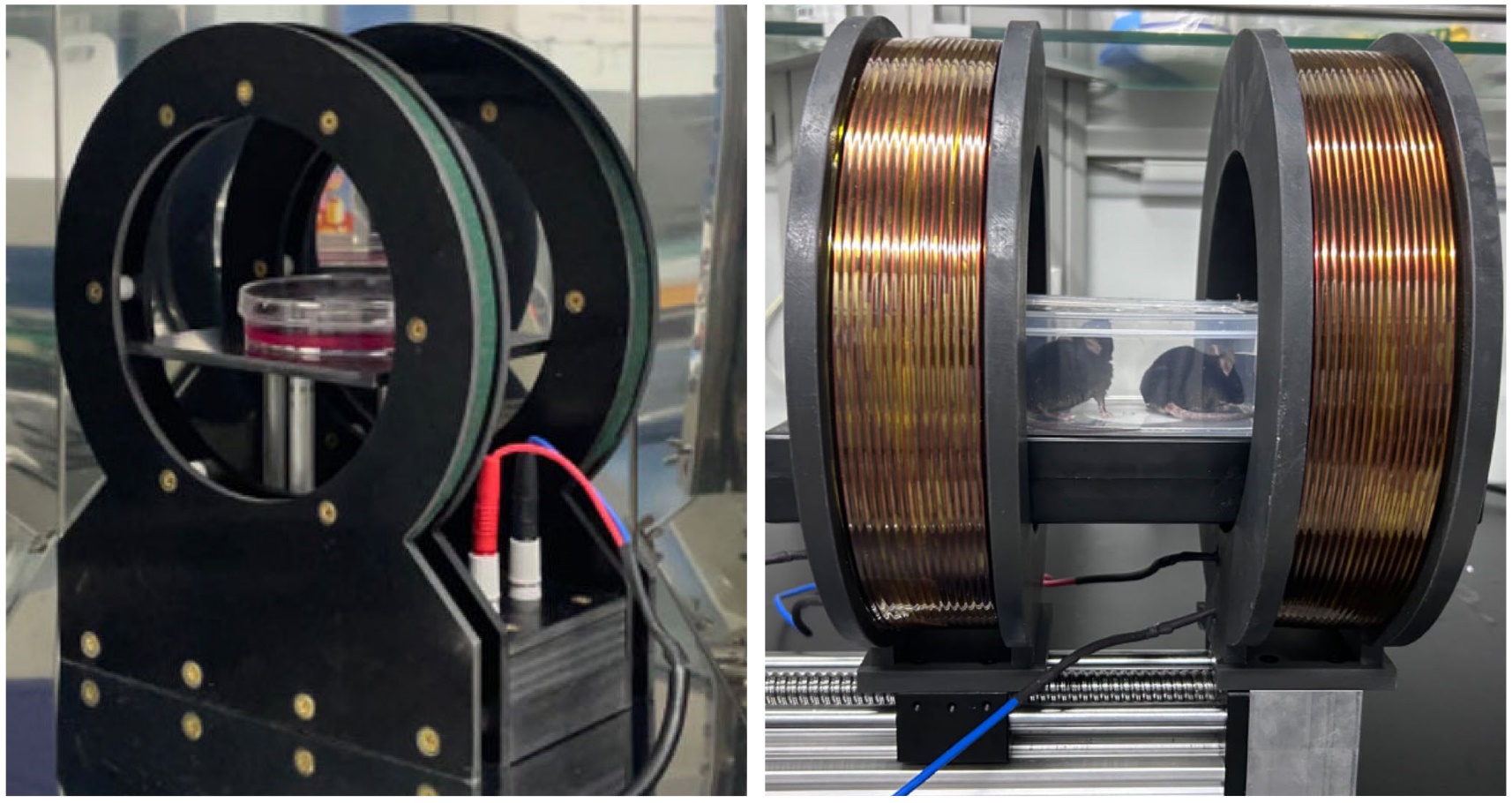


**Figure S1.** Images of two PEMF generators for cell culture and animal experiments. The intensities and frequencies of the PEMF can be controlled through a signal generator and power amplifier.


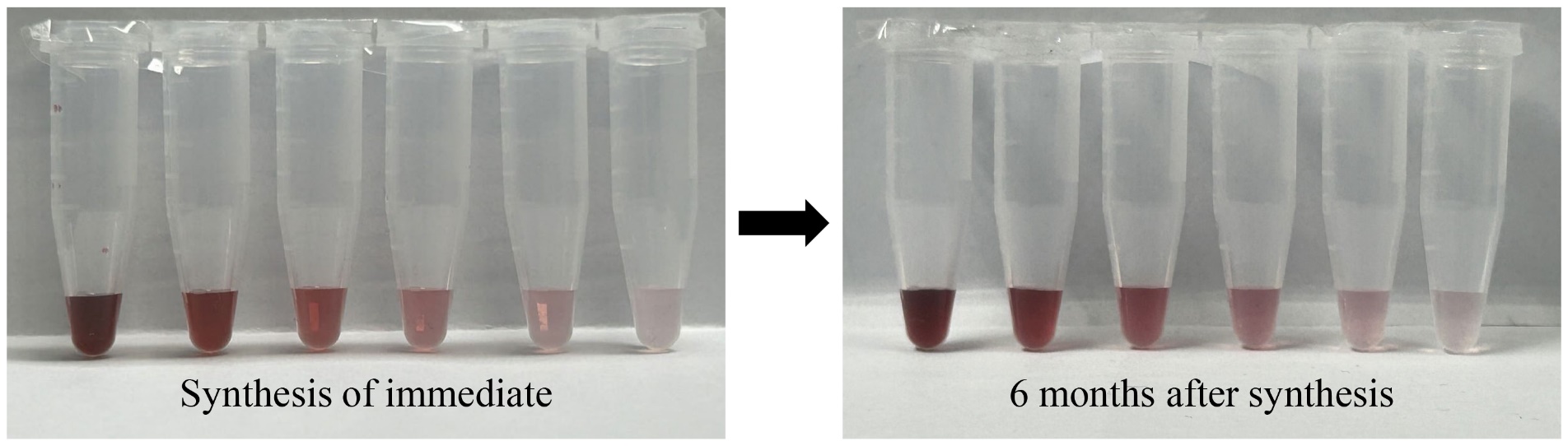


**Fig. S2.** PEG-AuNPs exhibited excellent long-term stability.


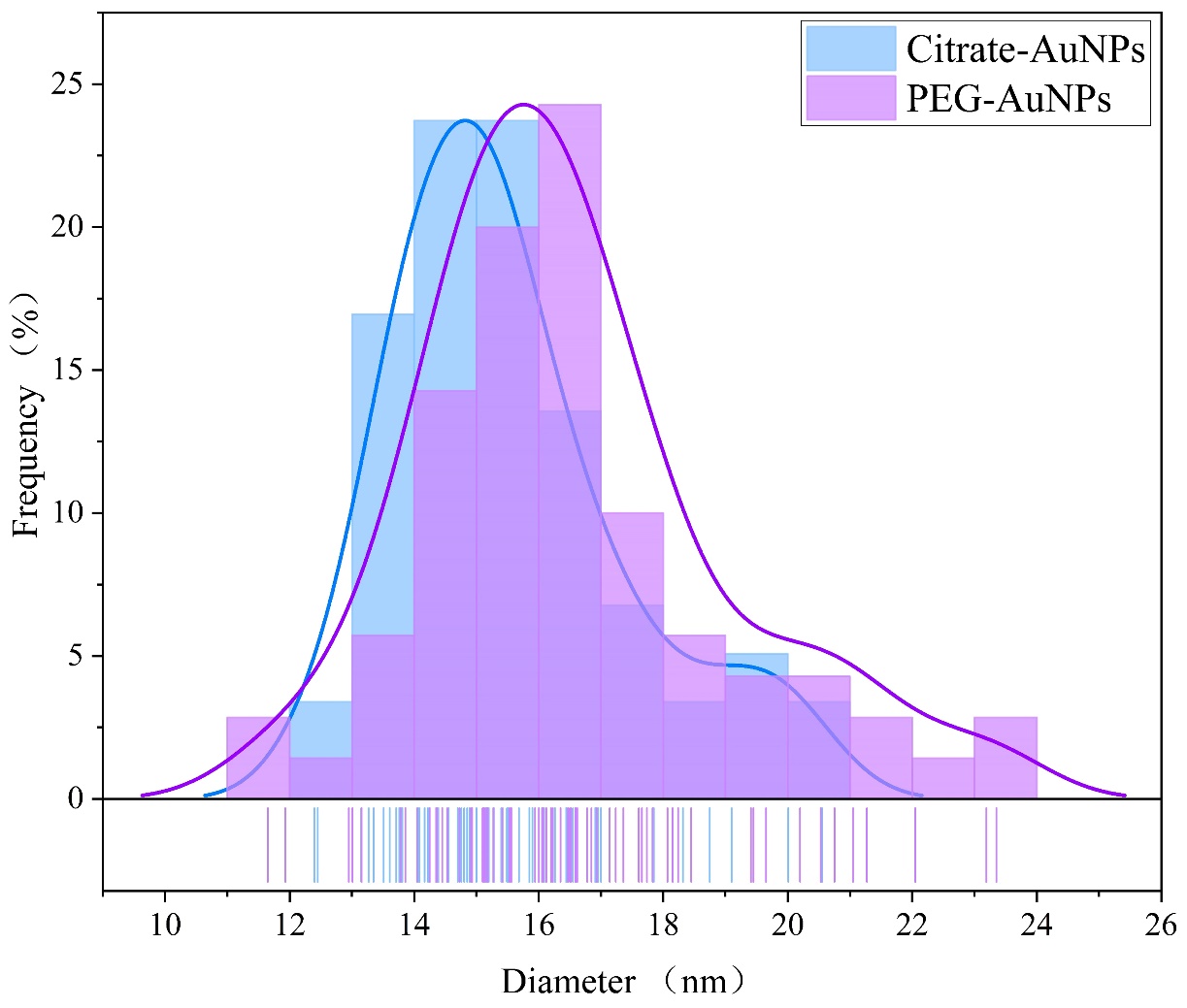


**Fig. S3.** Particle size distribution histogram of citrate-AuNPs and PEG-AuNPs.


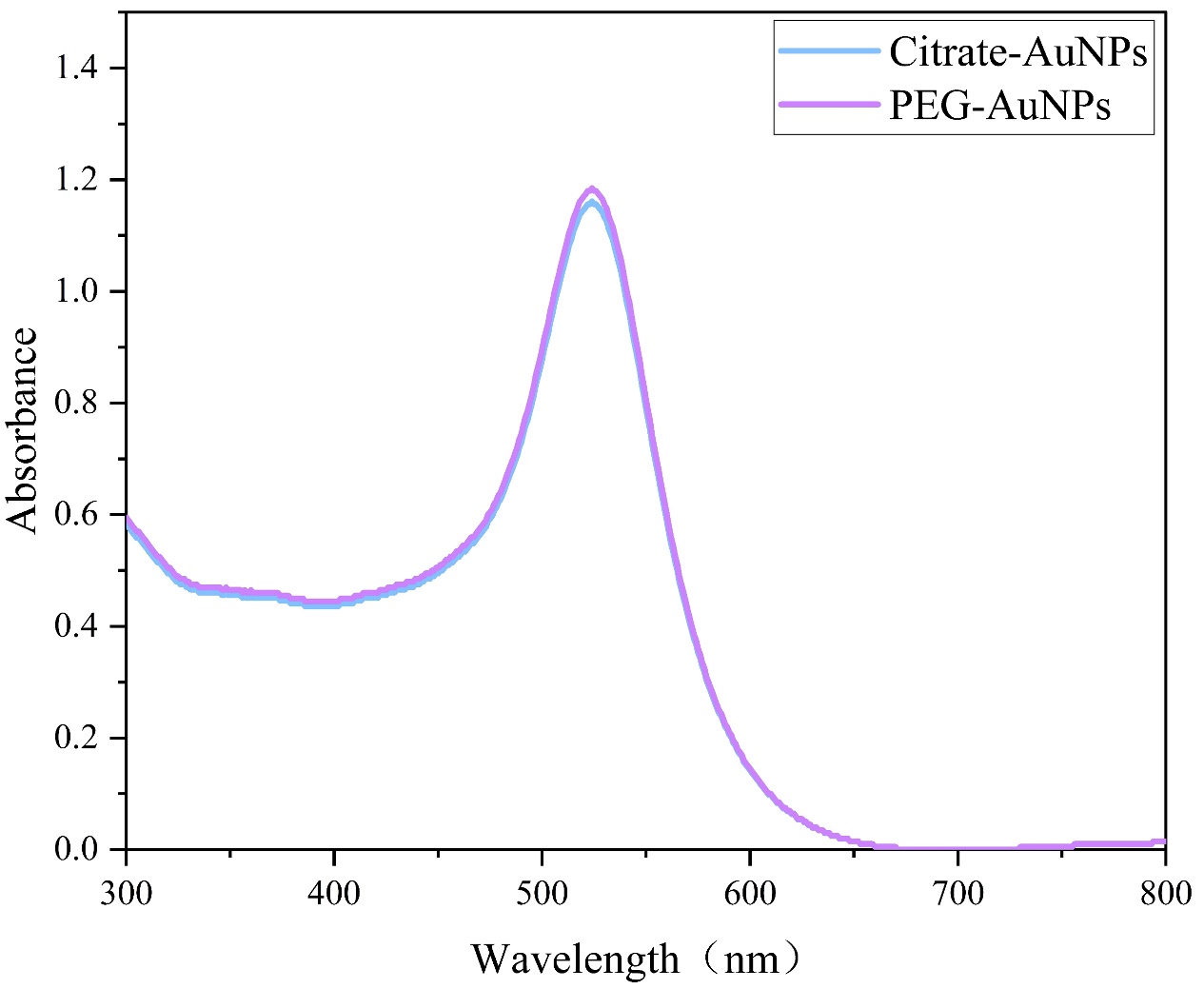


**Fig. S4.** UV-Vis absorption spectra of Citrate-AuNPs and PEG-AuNPs.


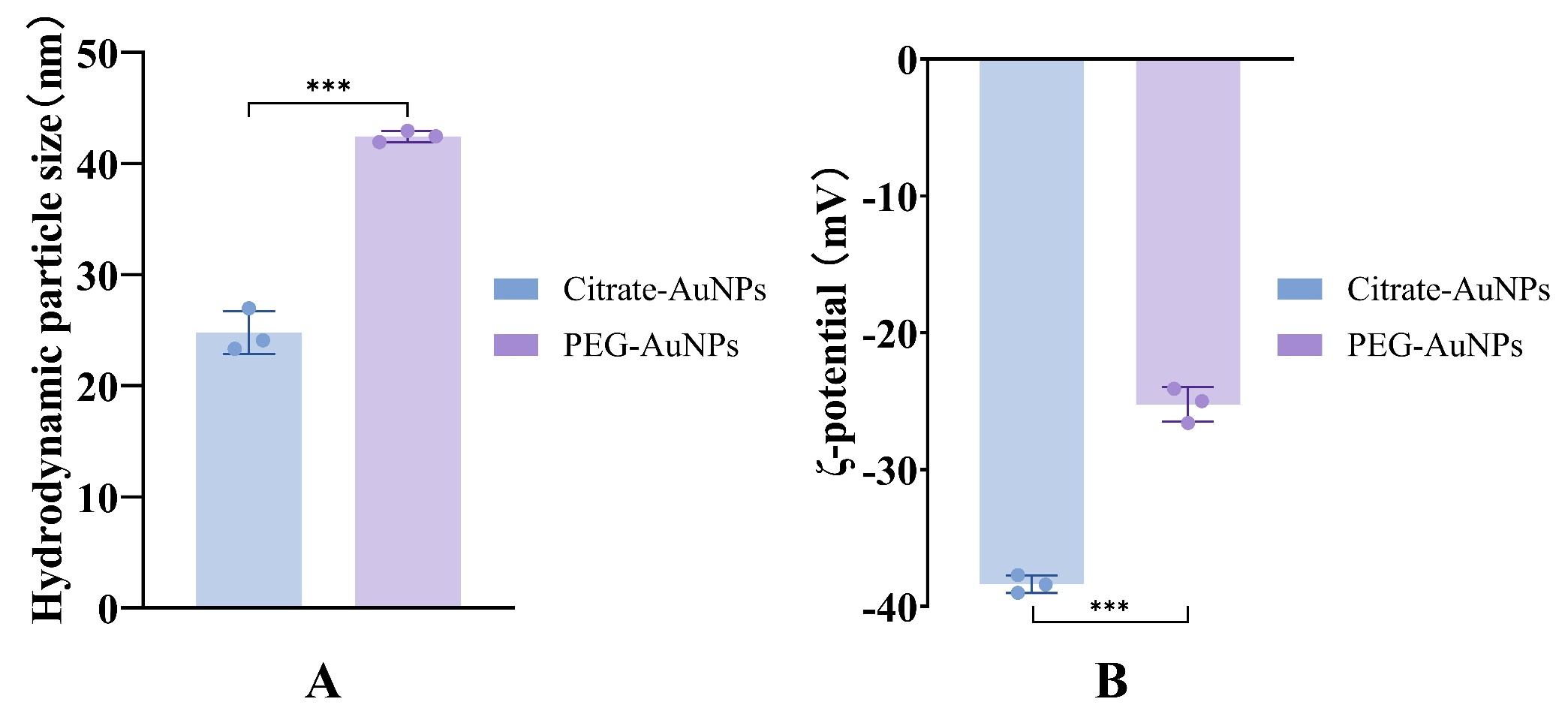


**Fig. S5.** The changes in average hydrodynamic particle size and ζ-potential after functionalization. (A) The change of average hydrodynamic particle size. (B) The change of average ζ-potential. A student’s t-test was conducted, with significance levels indicated as ****p* < 0.001.


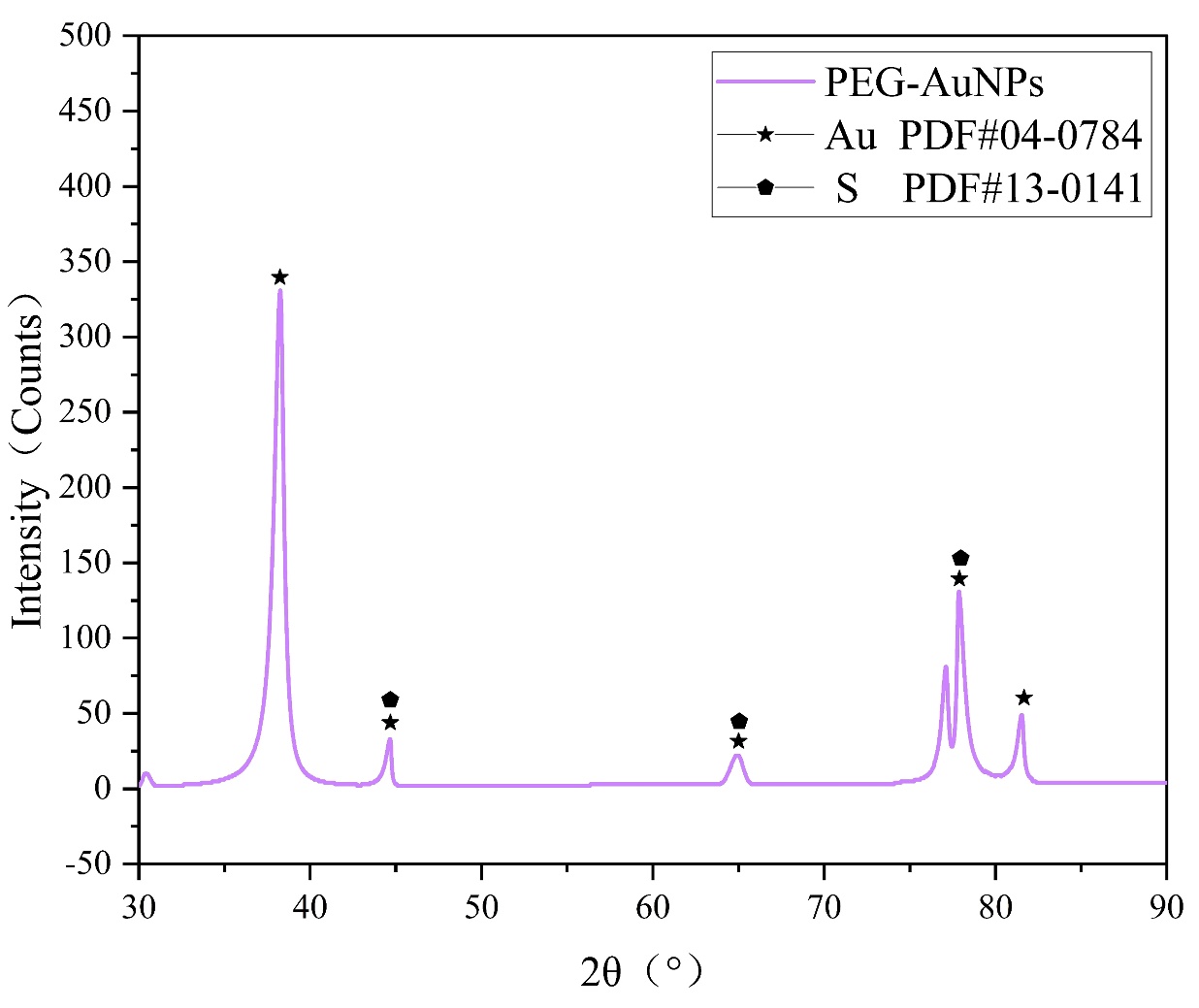


**Fig. S6.** The successful conjugation of the PEG groups onto the nanoparticles was confirmed by XRD.


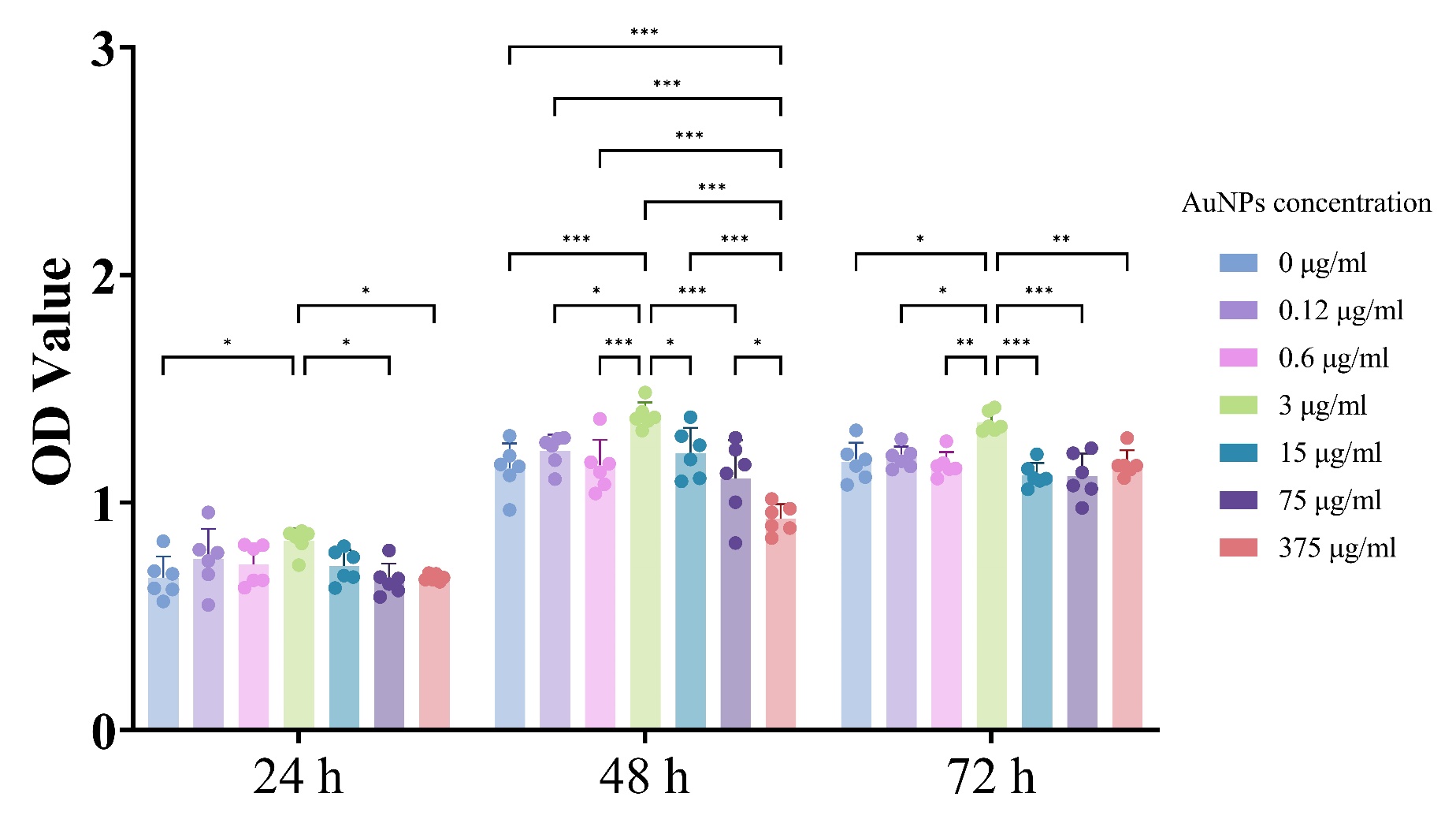


**Fig. S7.** Cell vitality following treatment with different concentrations of AuNPs. A two-way ANOVA was conducted, with significance levels indicated as **p* < 0.05, ***p* < 0.01, and ****p* < 0.001.





**Fig. S8.** Evaluating the distribution of effective field strength at various intensities of PEMF over distance using a Gauss meter. (A) *In vitro* coil. (B) *In vivo* coil.


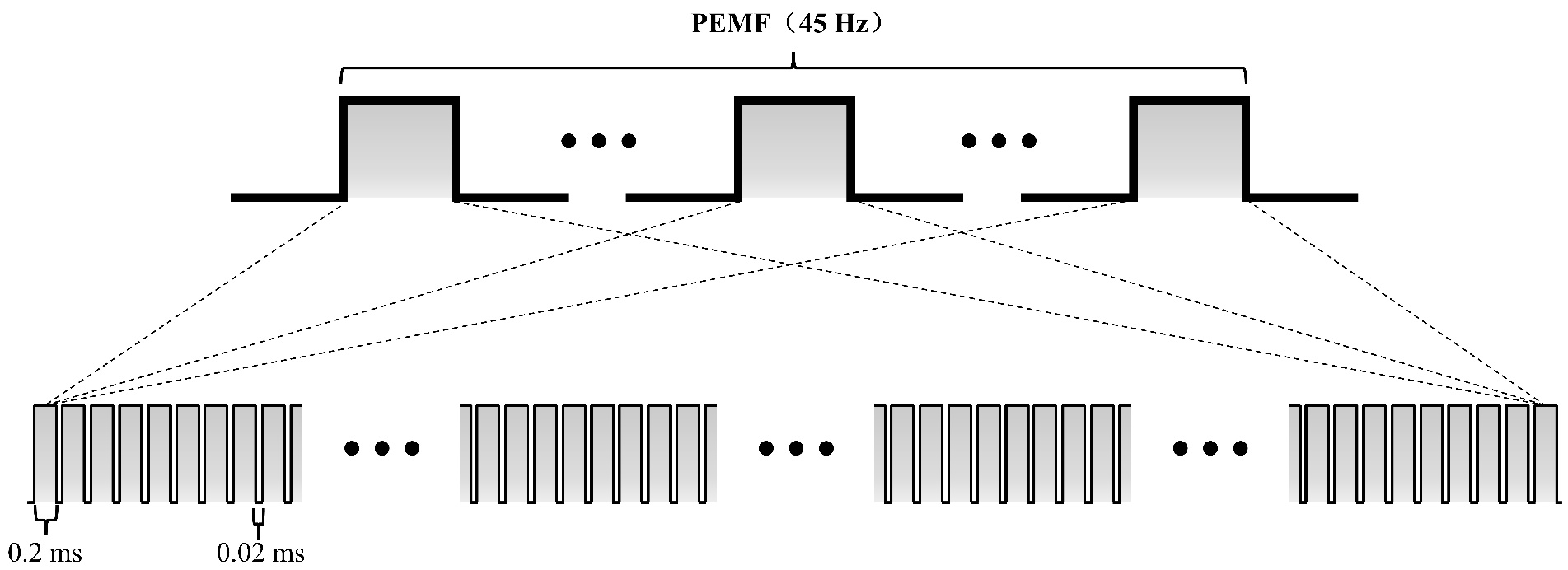


**Fig. S9.** Schematic illustrations displaying the waveform of PEMF. The PEMF waveform has a duty cycle of 50%, with a frequency of 45 Hz. This waveform is modulated by another carrier wave that has a duty cycle of 90.9% with a frequency of 4.545 kHz.


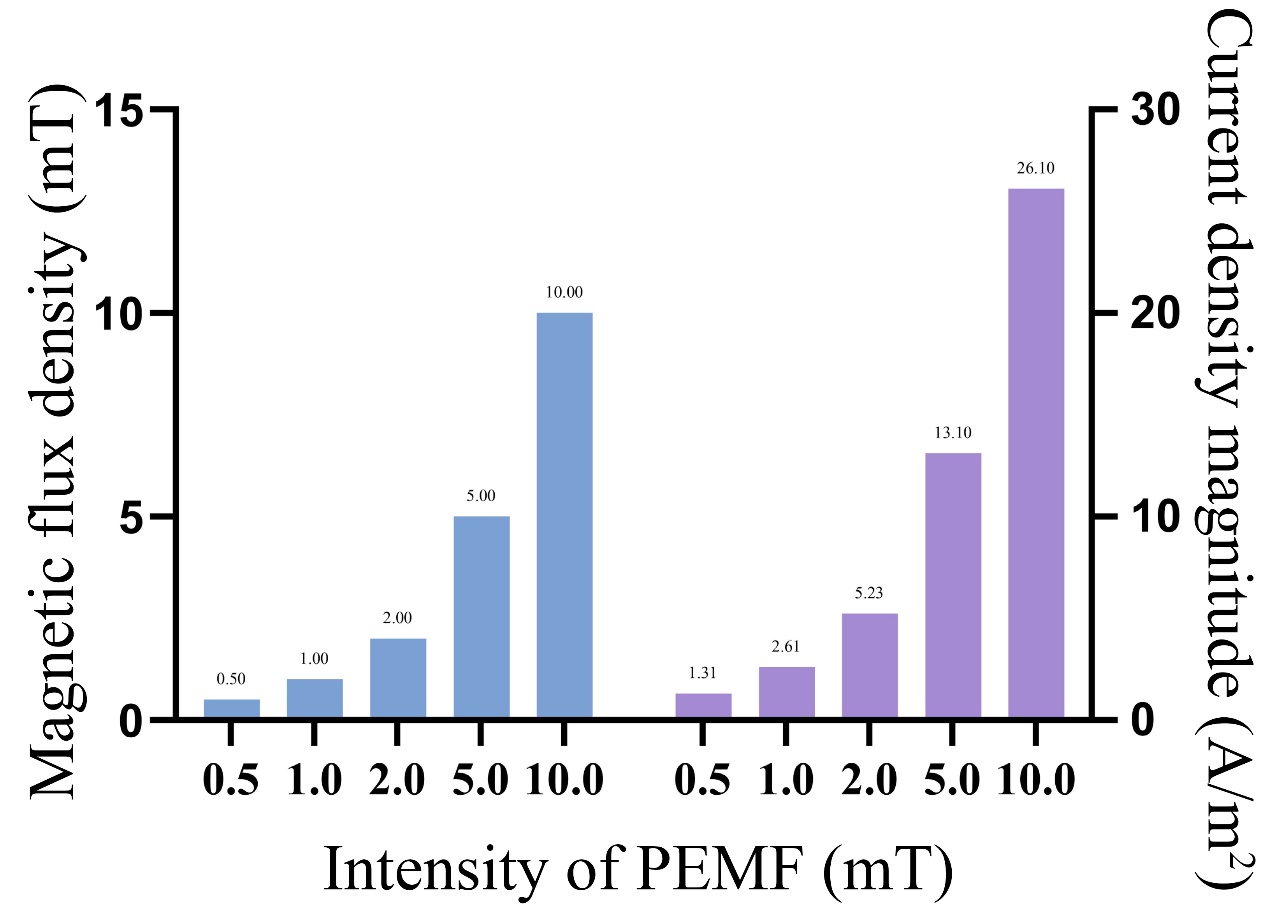


**Fig. S10.** The magnitude of the magnetic flux density and current density on the surface of AuNPs under different peak intensities of PEMF. Blue bars (left Y-axis) represent the magnetic flux density (mT) and purple bars (right Y-axis) indicate the current density (A/m^2^) on the surface of AuNPs under various PEMF intensities, as determined by COMSOL simulations. Both parameters show a gradual increase with increasing PEMF strength.


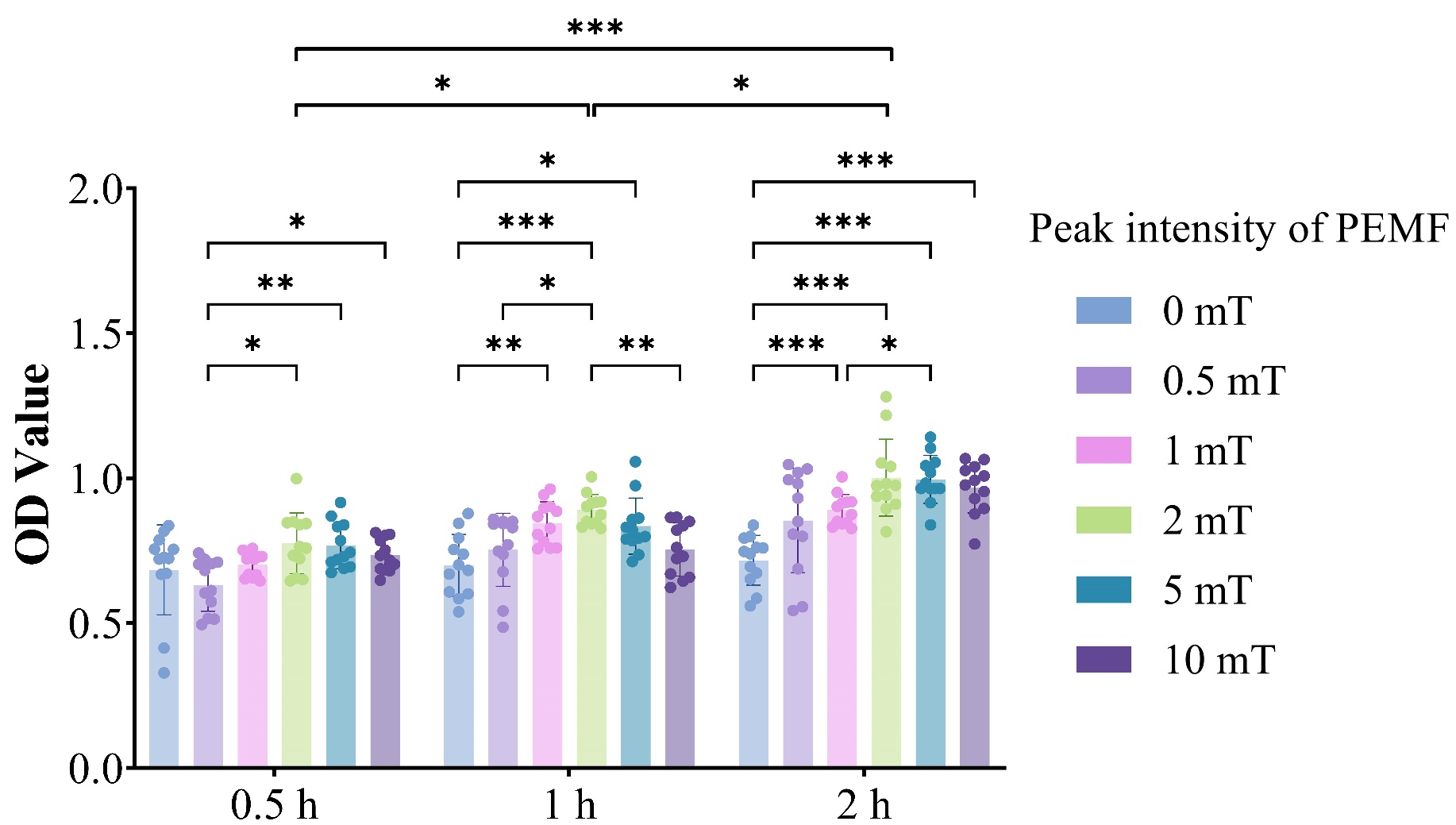


**Fig. S11.** Cell vitality following exposure to various durations and intensities of PEMF. Short-duration stimulation (0.5 h) showed limited effects, while prolonged exposure (1 and 2 h) led to a time-dependent increase in viability. The most pronounced enhancement was observed at 2 mT for 2 h, whereas higher intensities (5 and 10 mT) showed a decreasing trend. A two-way ANOVA was conducted, with significance levels indicated as **p* < 0.05, ***p* < 0.01, and ****p* < 0.001.


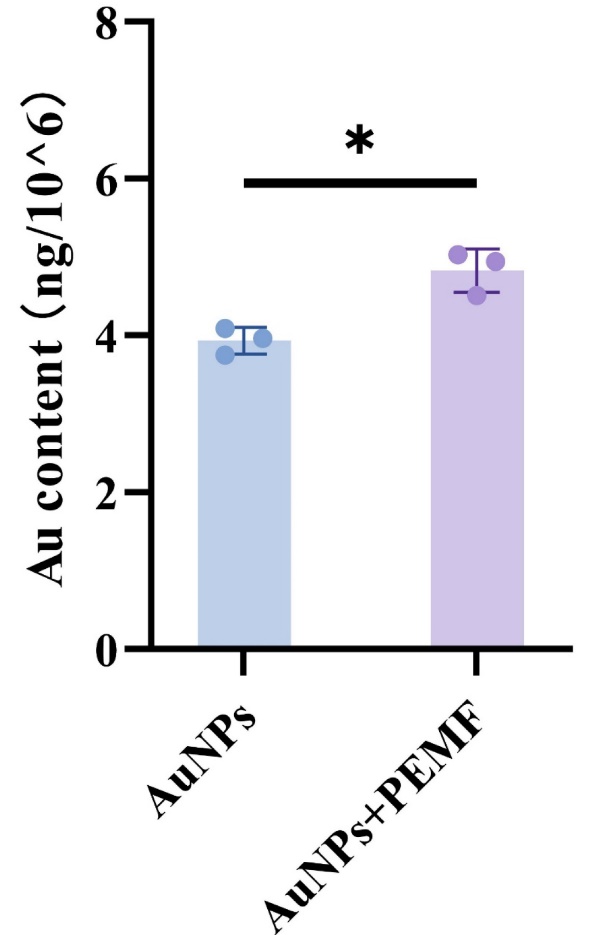


**Fig. S12.** The amount of AuNPs internalized by MC3T3-E1 cells with and without PEMF exposure was quantified through inductively coupled plasma mass spectrometry. A student’s t-test was conducted, with significance levels indicated as **p* < 0.05.


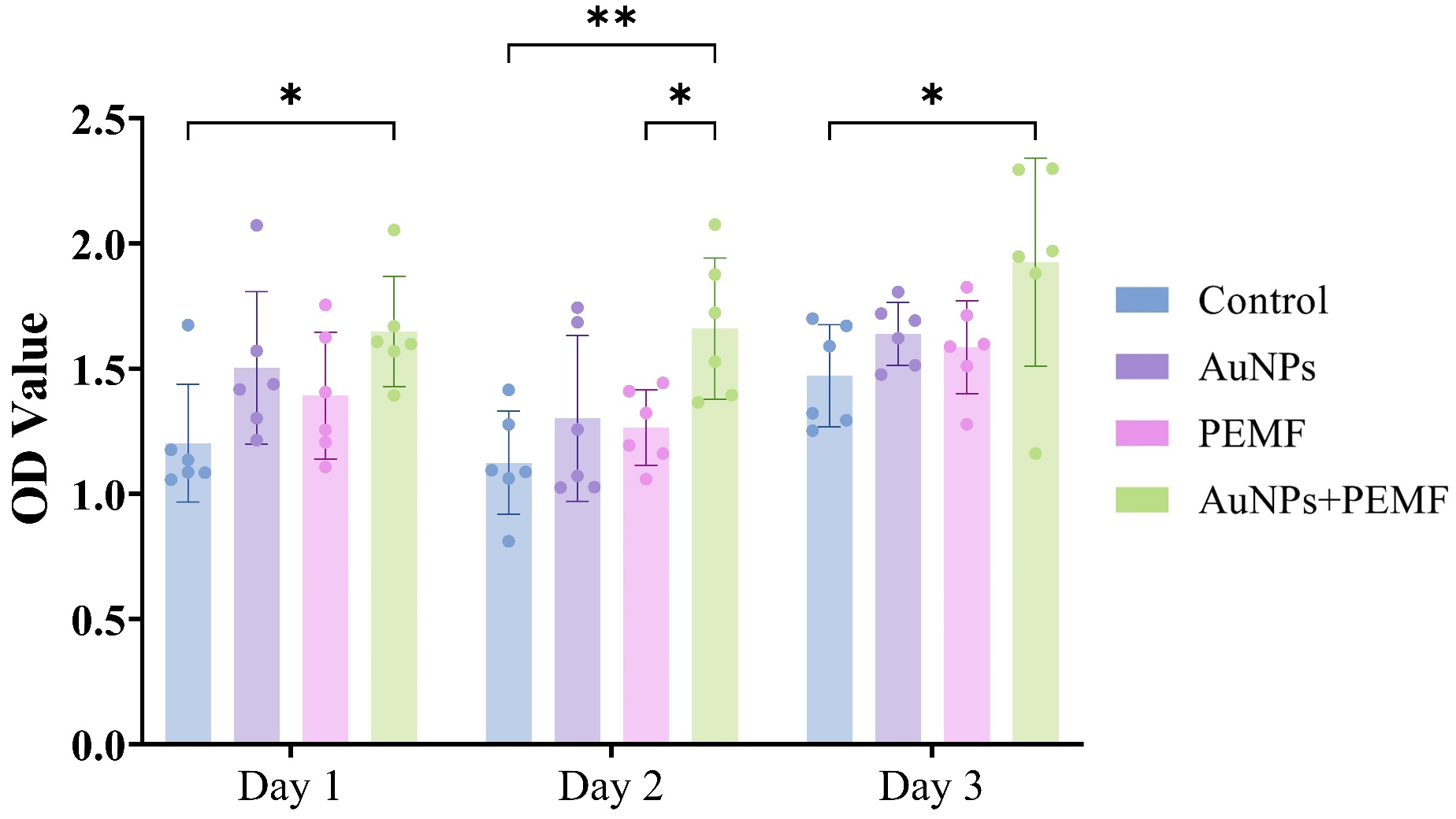


**Fig. S13.** Cell viability after the treatment of electromagnetized AuNPs as measured by CCK-8 assay. A two-way ANOVA was conducted, with significance levels indicated as **p* < 0.05, and ***p* < 0.01.


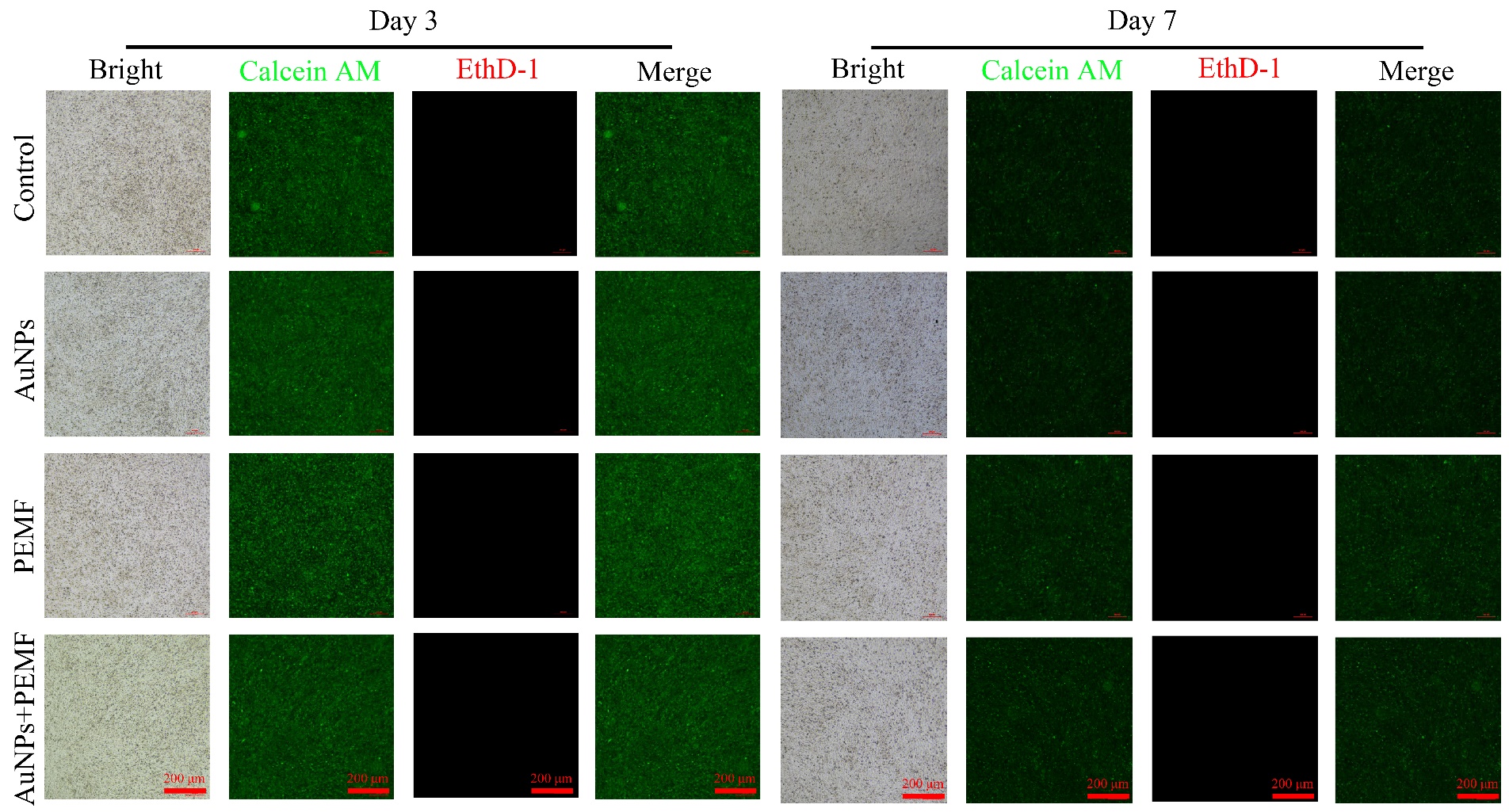


**Fig. S14.** Live/dead cell double staining was performed after different treatments. Dead cells were stained red while living cells were stained green.


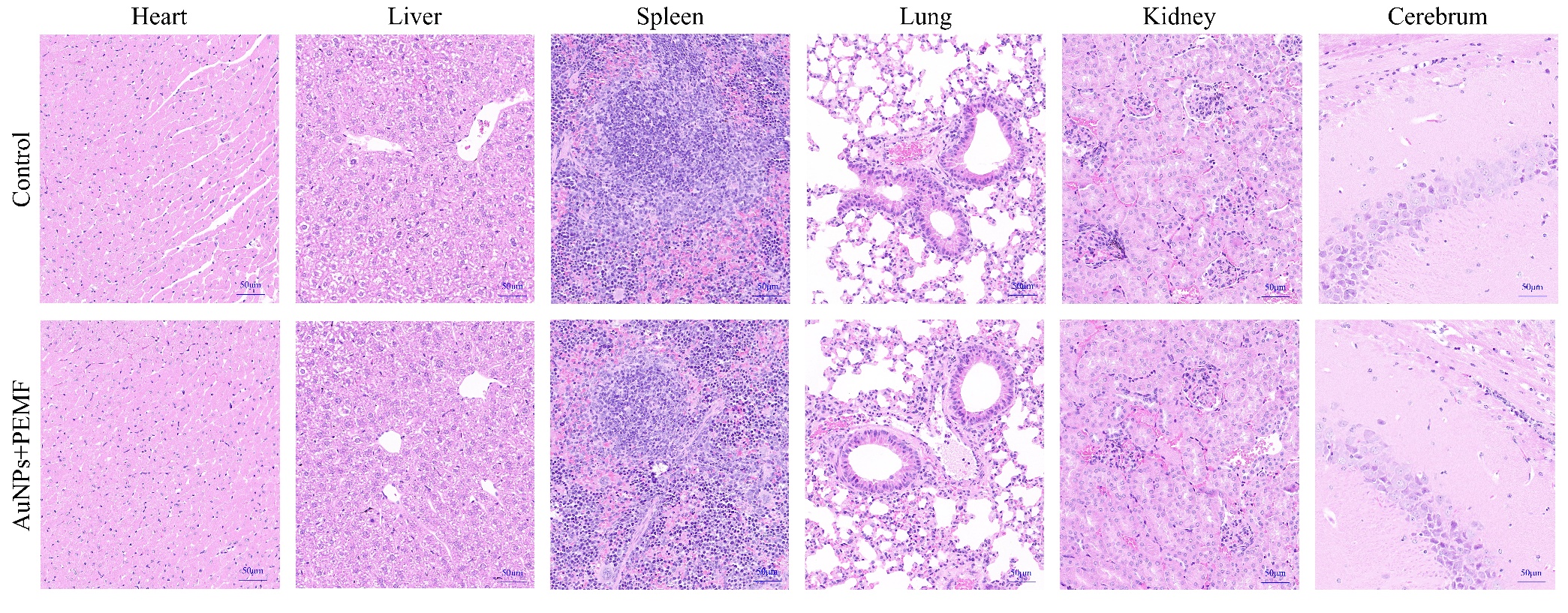


**Fig. S15.** Representative H&E staining of the heart, liver, spleen, lungs, kidneys, and cerebrum following 2 weeks of treatment with electromagnetized AuNPs.


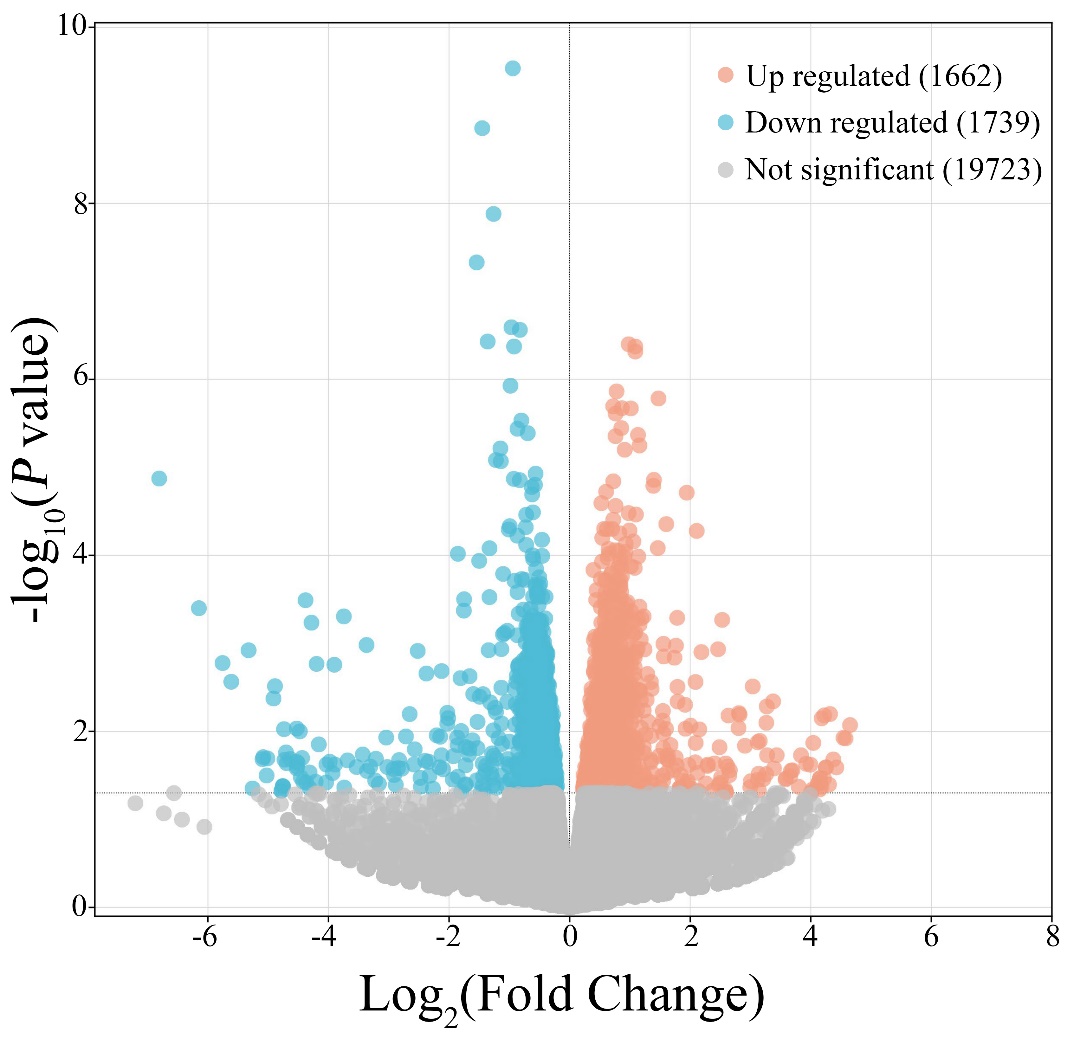


**Fig.S16.** Volcano plot of differentially expressed genes after treatment with electromagnetized AuNPs, with red indicating upregulation and blue indicating downregulation.


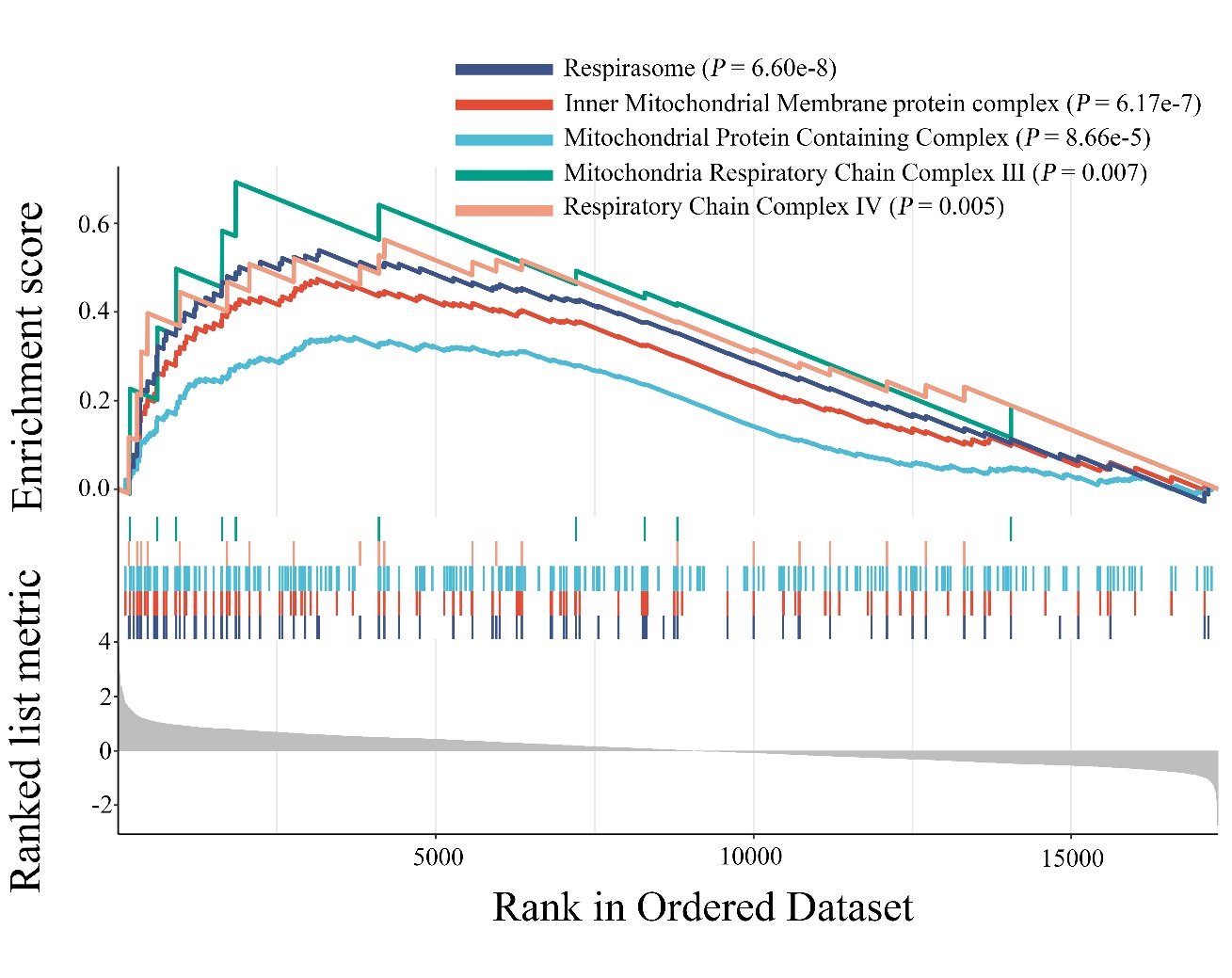


**Fig. S17.** GSEA of GO-CC show a significant upregulation of mitochondrial function gene sets in the electromagnetized AuNPs-treated group.


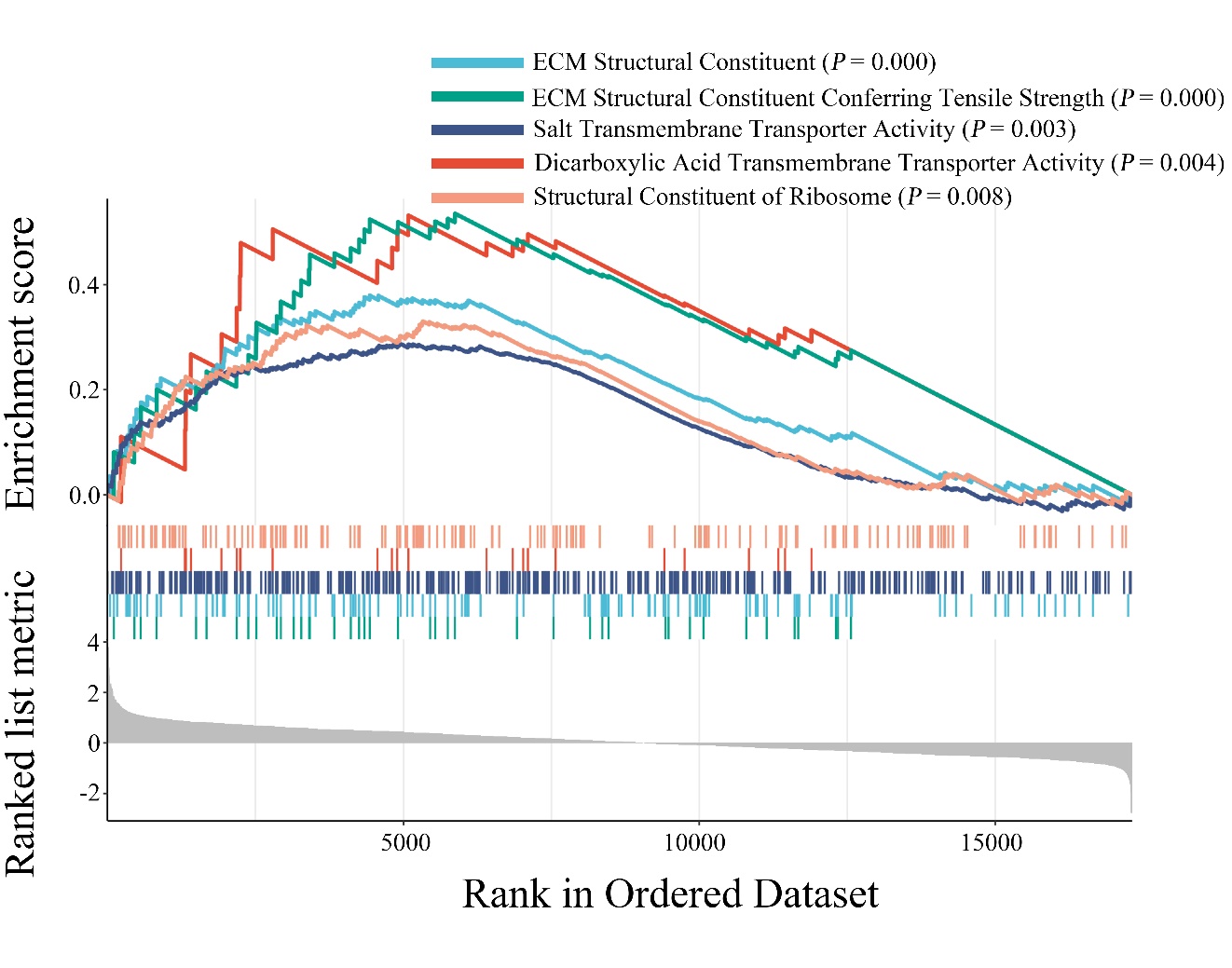


**Fig. S18.** GSEA of GO-MF illustrated a significant upregulation of extracellular matrix structure gene sets in the electromagnetized AuNPs-treated group.


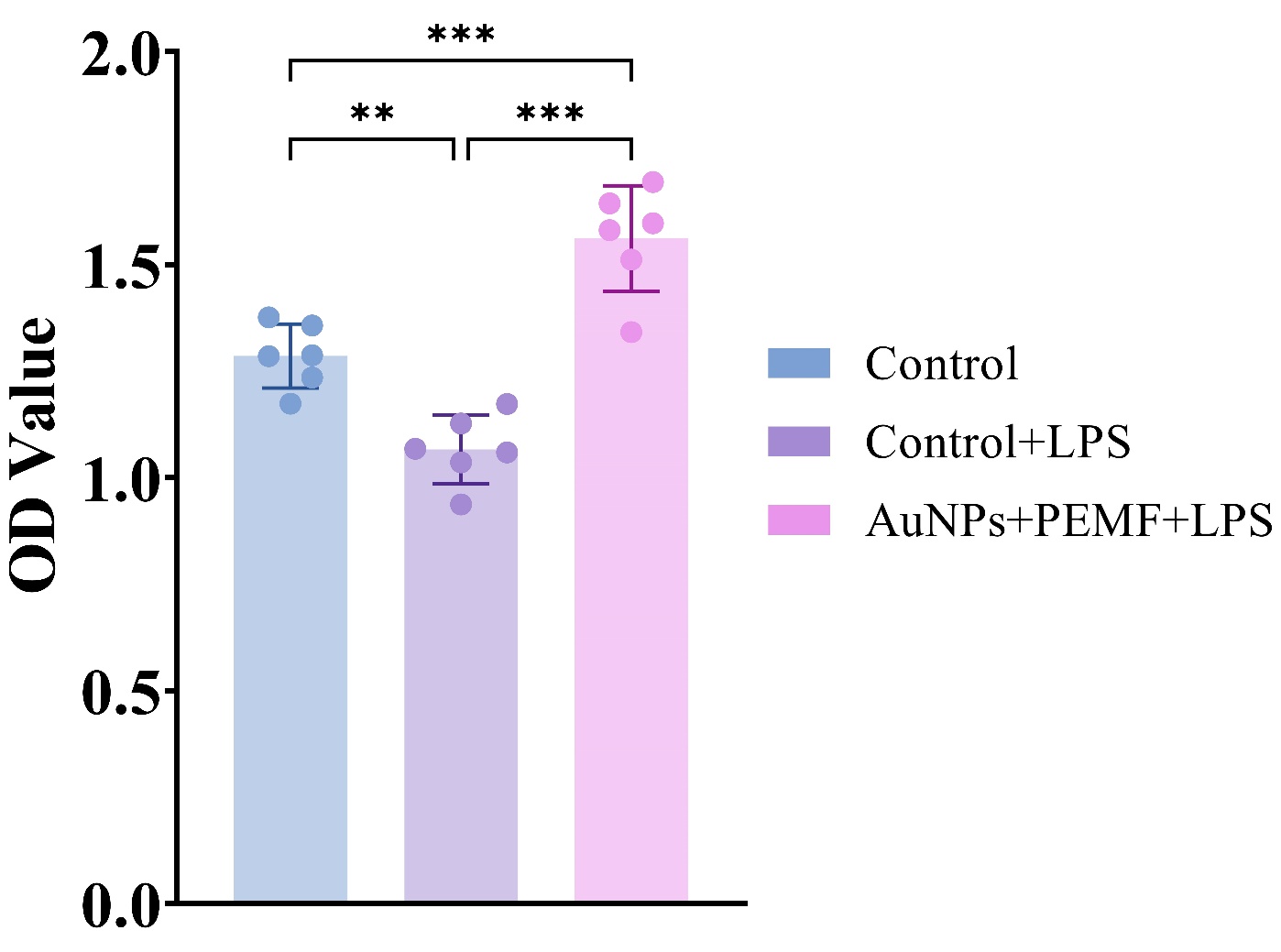


**Fig. S19.** Cell viability of MC3T3-E1 cells under inflammatory conditions following various treatments, as measured by CCK-8 assay. A one-way ANOVA was conducted, with significance levels indicated as ***p* < 0.01, and ****p* < 0.001.


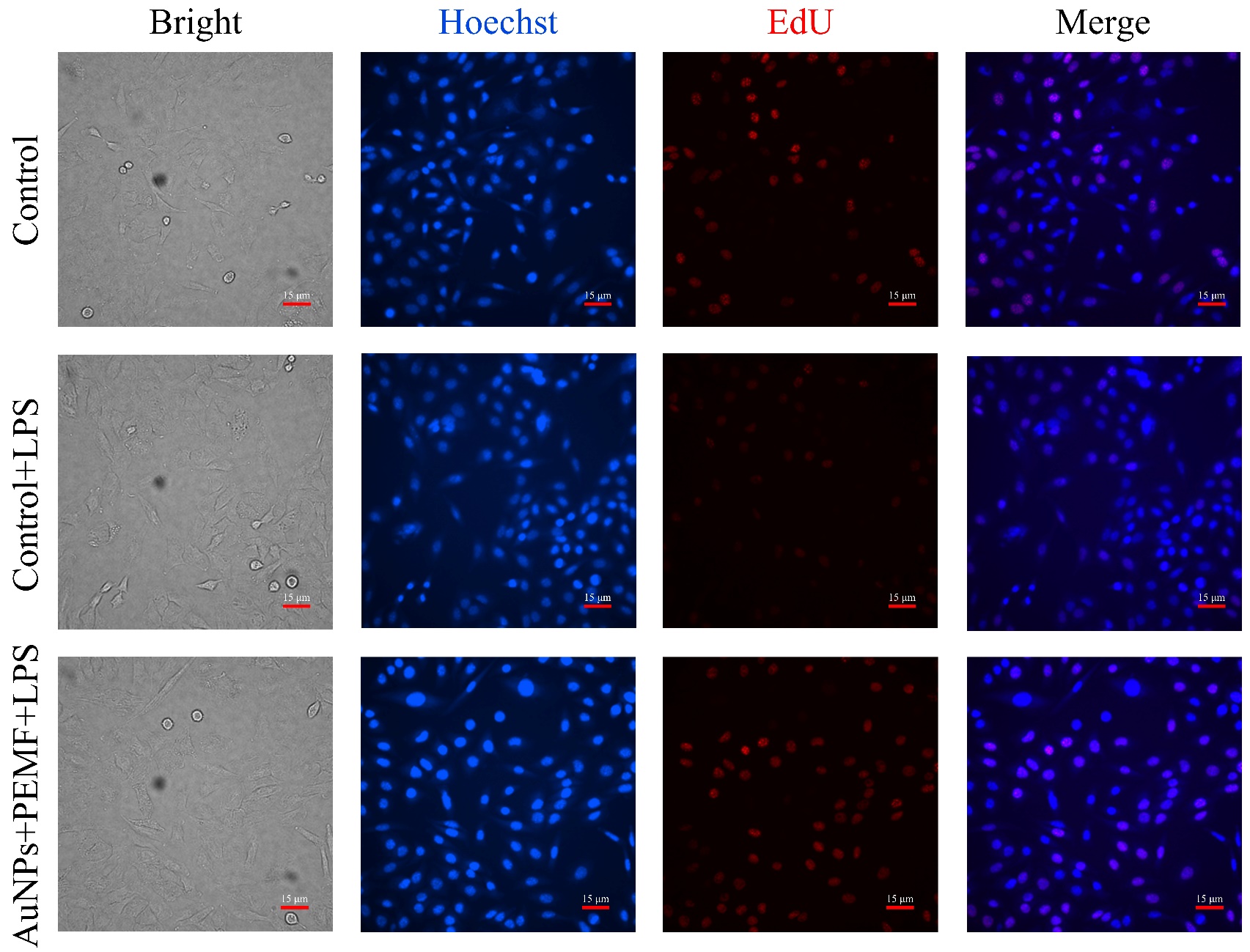


**Fig. S20.** Representative images of EdU assay.


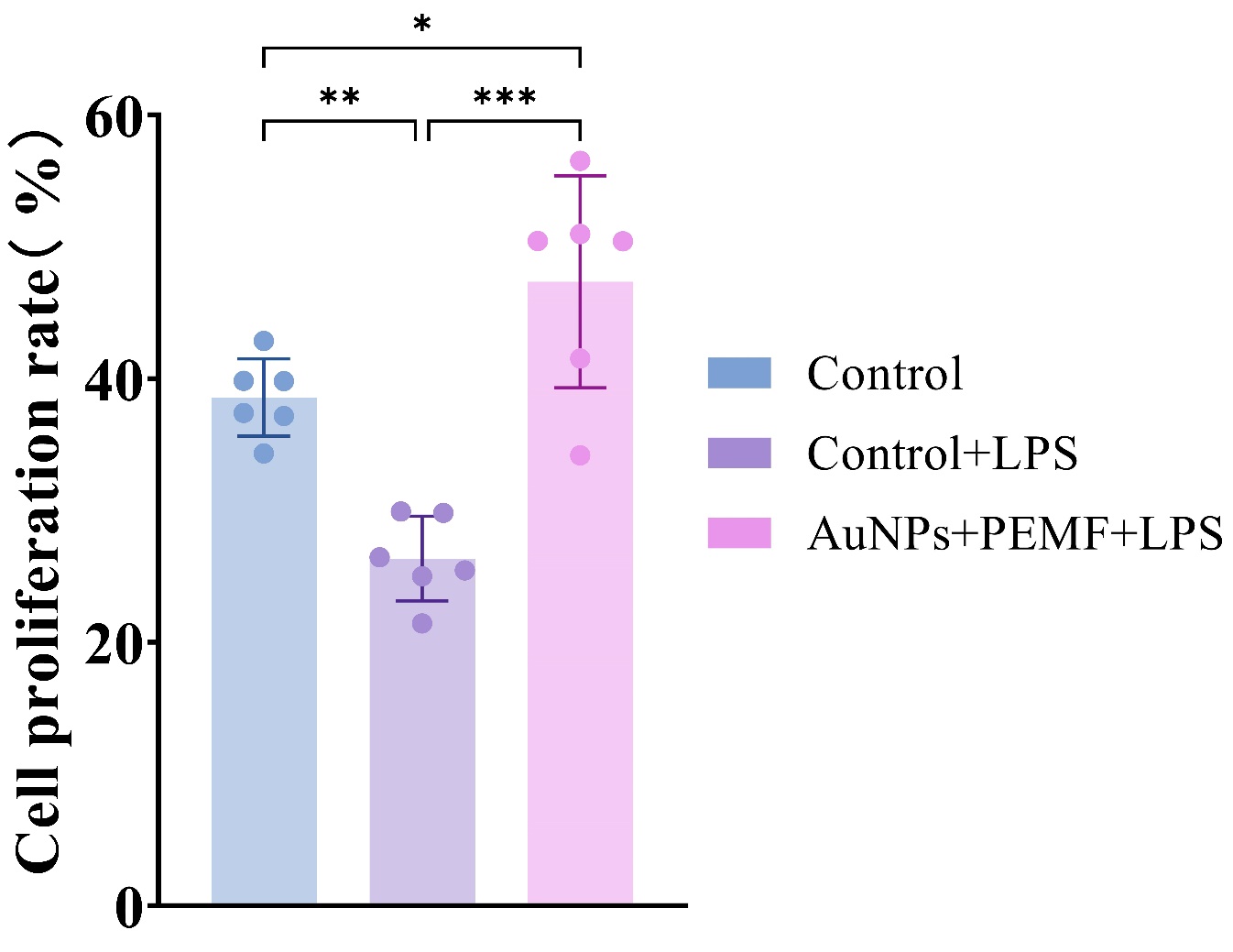


**Fig. S21.** Quantitative analysis of EdU assay. A one-way ANOVA was conducted, with significance levels indicated as **p* < 0.05, ***p* < 0.01, and ****p* < 0.001.


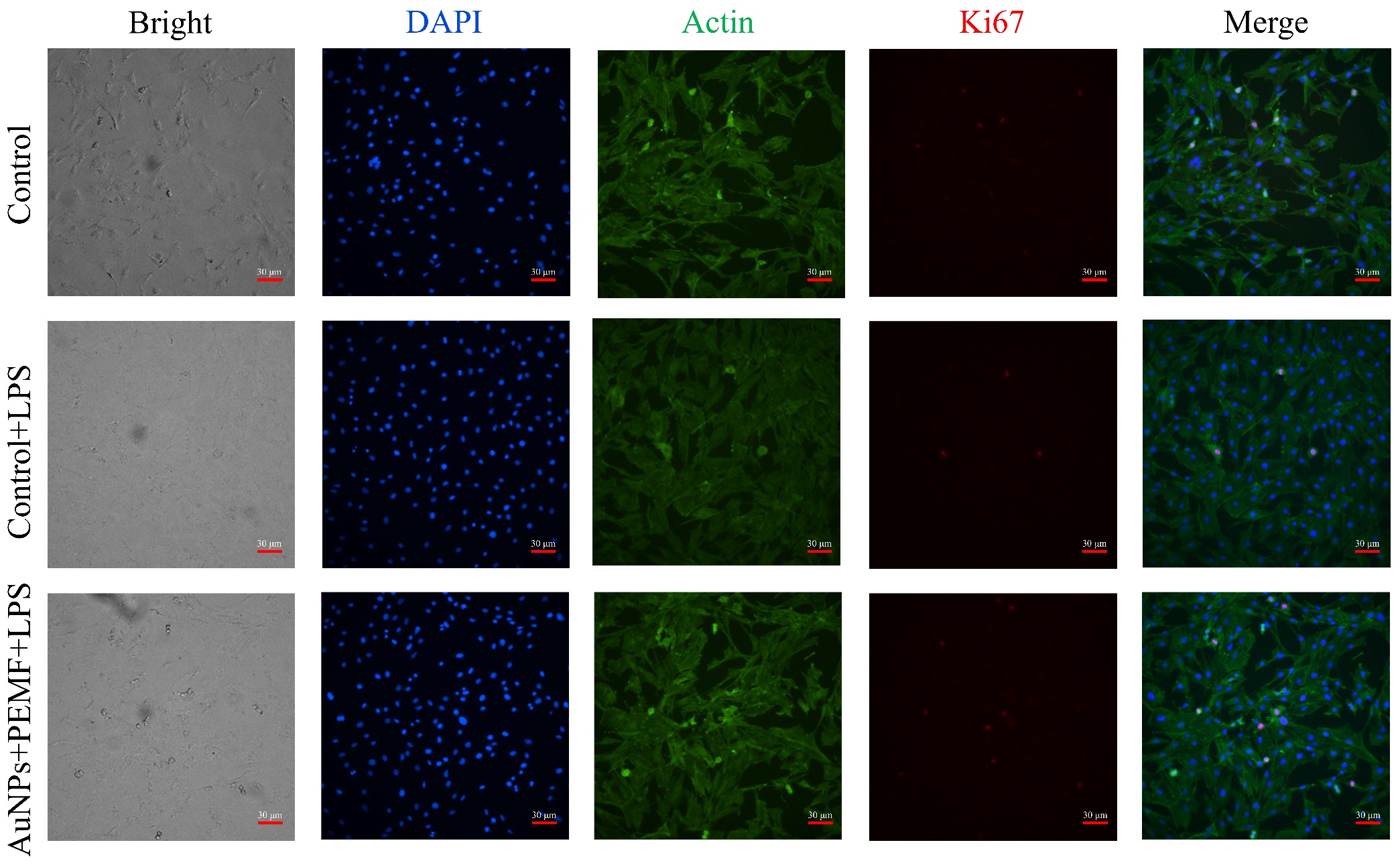


**Fig. S22.** Representative images of Ki67 protein IF staining.


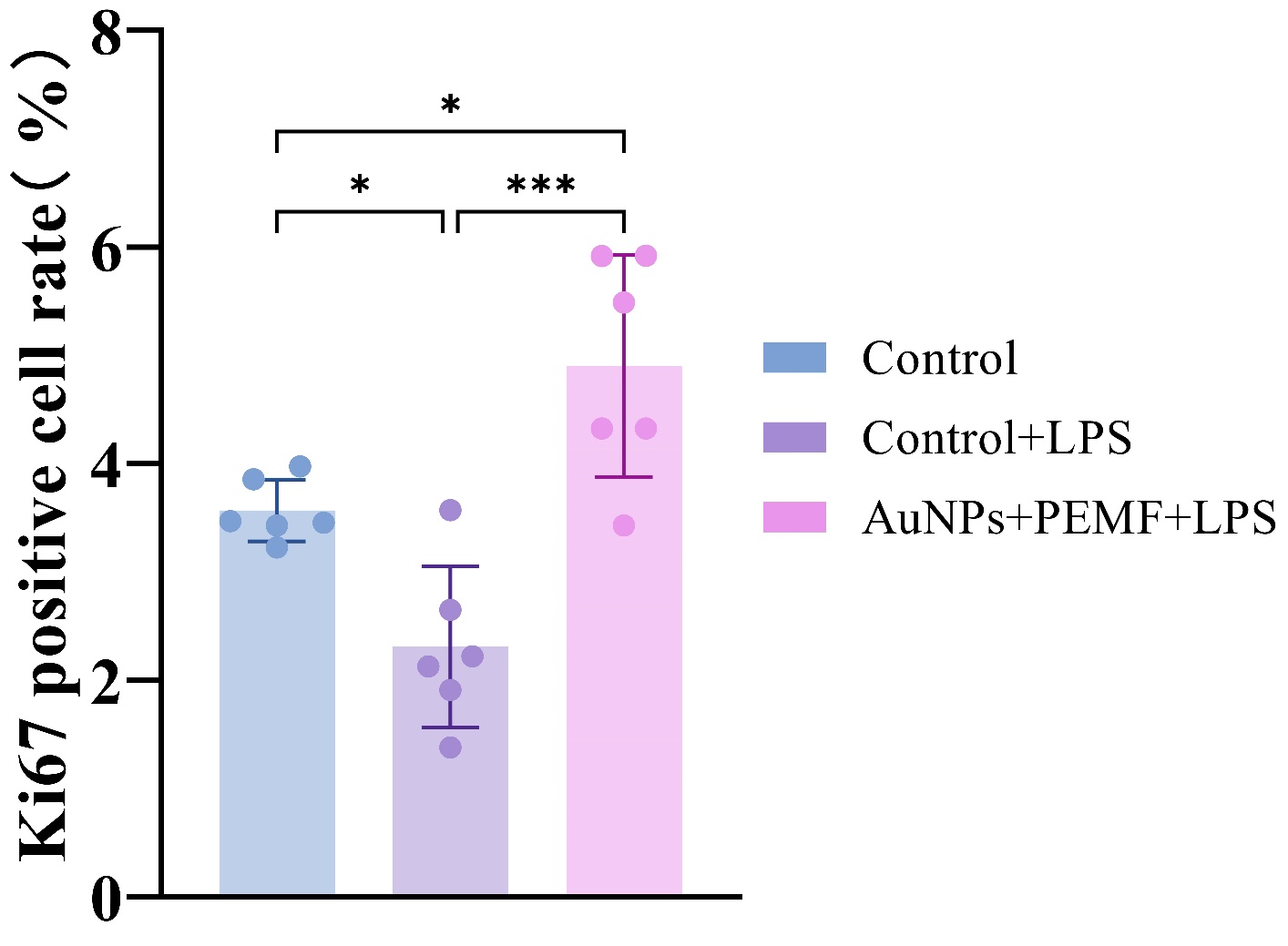


**Fig. S23.** Quantitative analysis of Ki67 protein IF staining. A one-way ANOVA was conducted, with significance levels indicated as **p* < 0.05, ***p* < 0.01, and ****p* < 0.001.


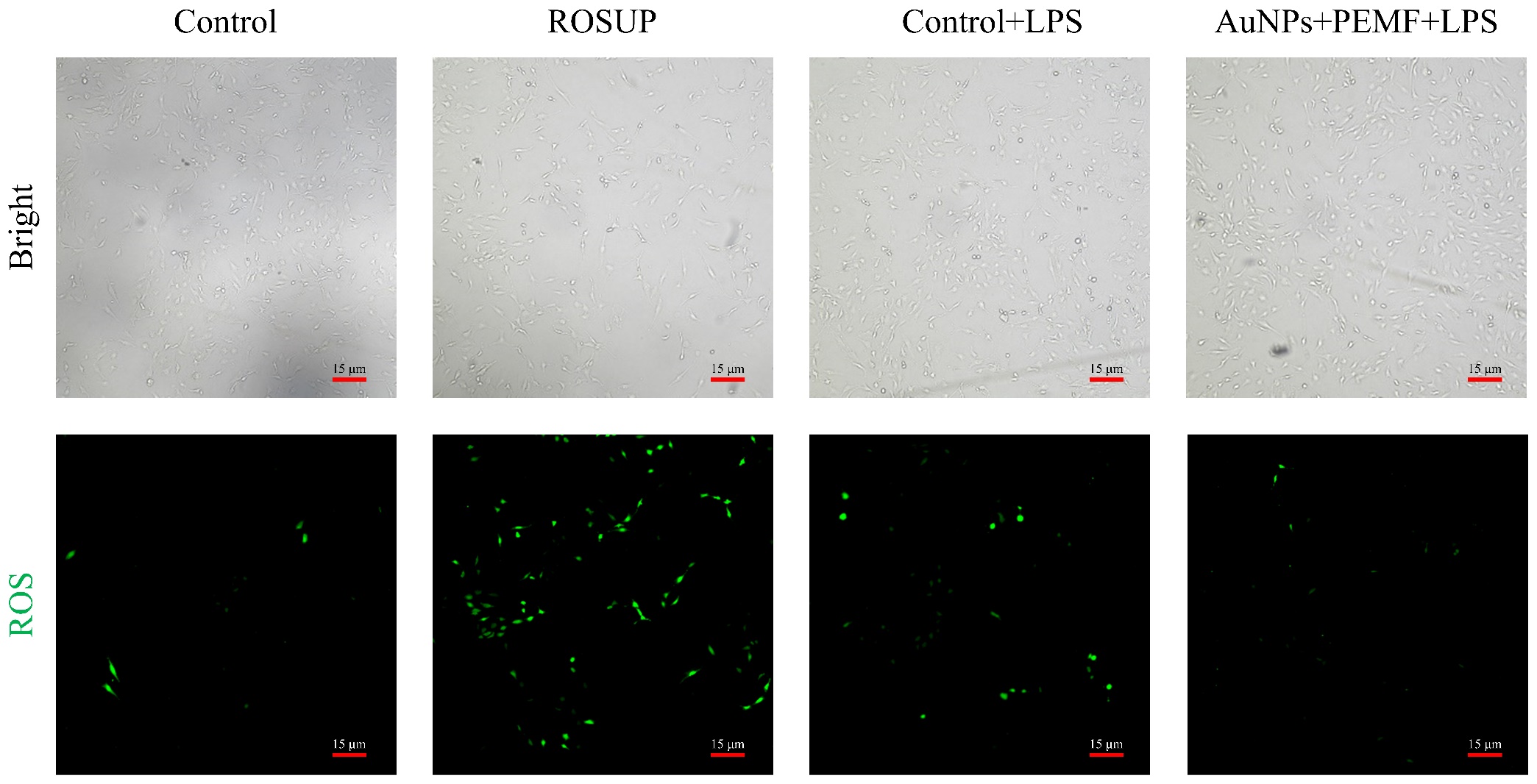


**Fig. S24.** Representative images and quantitative analysis of the ROS levels, as measured using the DCFH-DA fluorescent probe method.


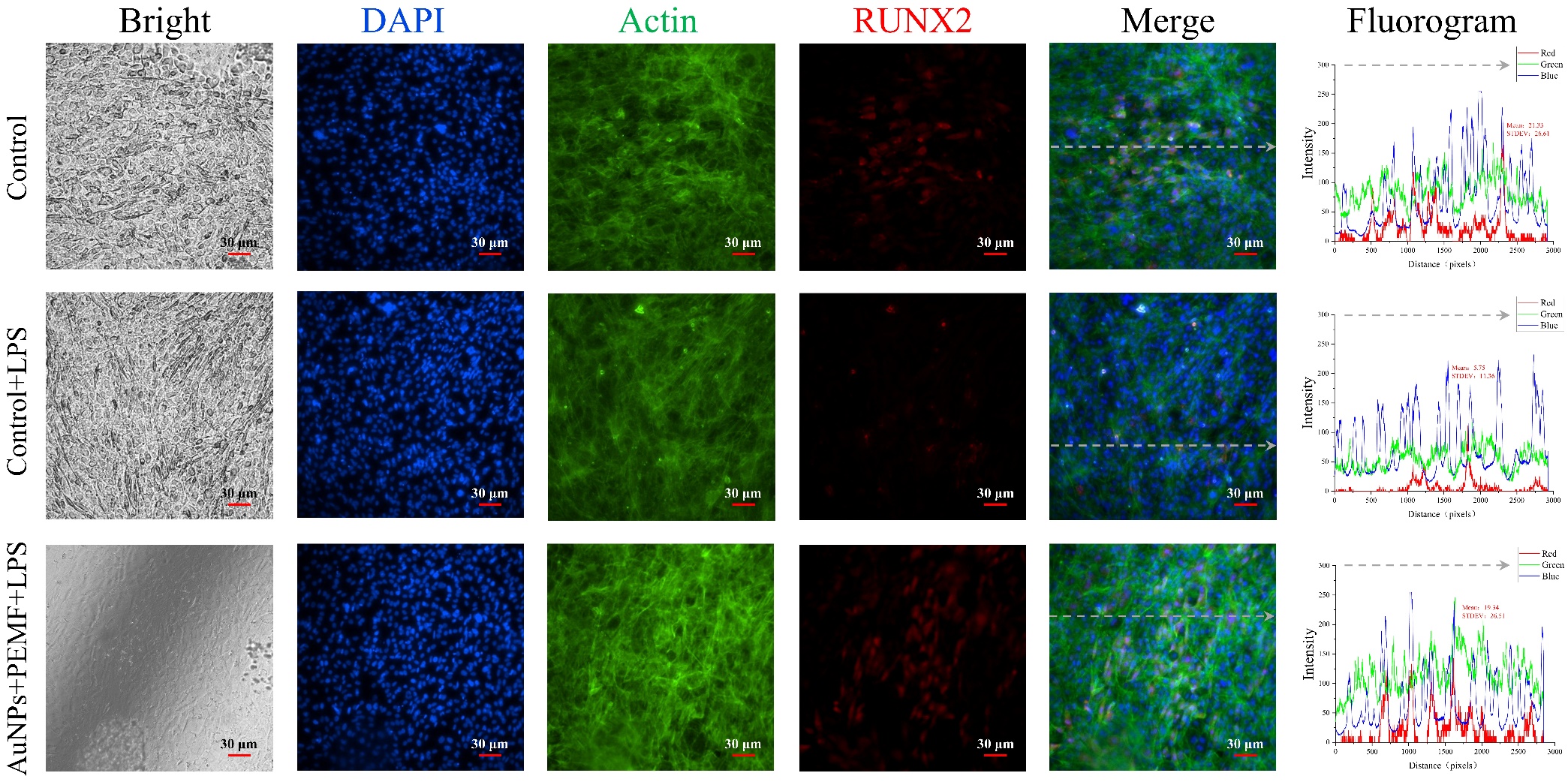


**Fig. S25.** Representative images and fluorescence intensity of RUNX2 protein IF staining.


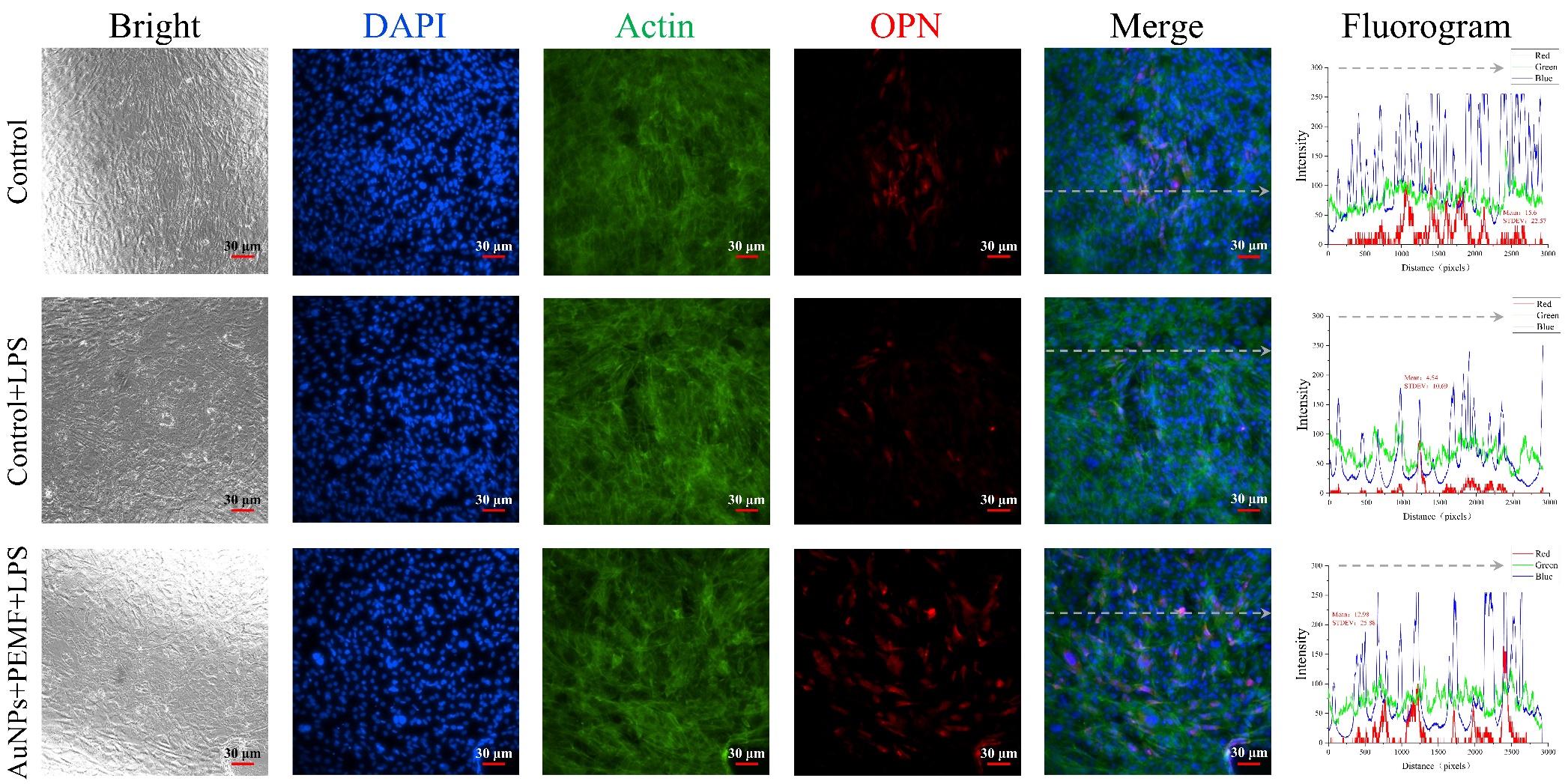


**Fig. S26.** Representative images and fluorescence intensity of OPN protein IF staining.





**Fig. S27.** Representative Micro-CT and 3D reconstruction images of each group.





**Fig. S28.** Quantitative analysis of the representative morphometric parameters of bone trabecula, as measured by Micro-CT. (A) Bone mineral density (BMD). (B) Percent bone volume (BV/TV). A one-way ANOVA was performed, with significance levels indicated as **p* < 0.05, ***p* < 0.01, and ****p* < 0.001.





**Fig. S29.** Quantitative RT-qPCR analysis of IL-17A, IL-17F, and IL-17RA mRNA expression in femoral bone tissue. A one-way ANOVA was performed, with significance levels indicated as **p* < 0.05, ***p* < 0.01, and ****p* < 0.001.


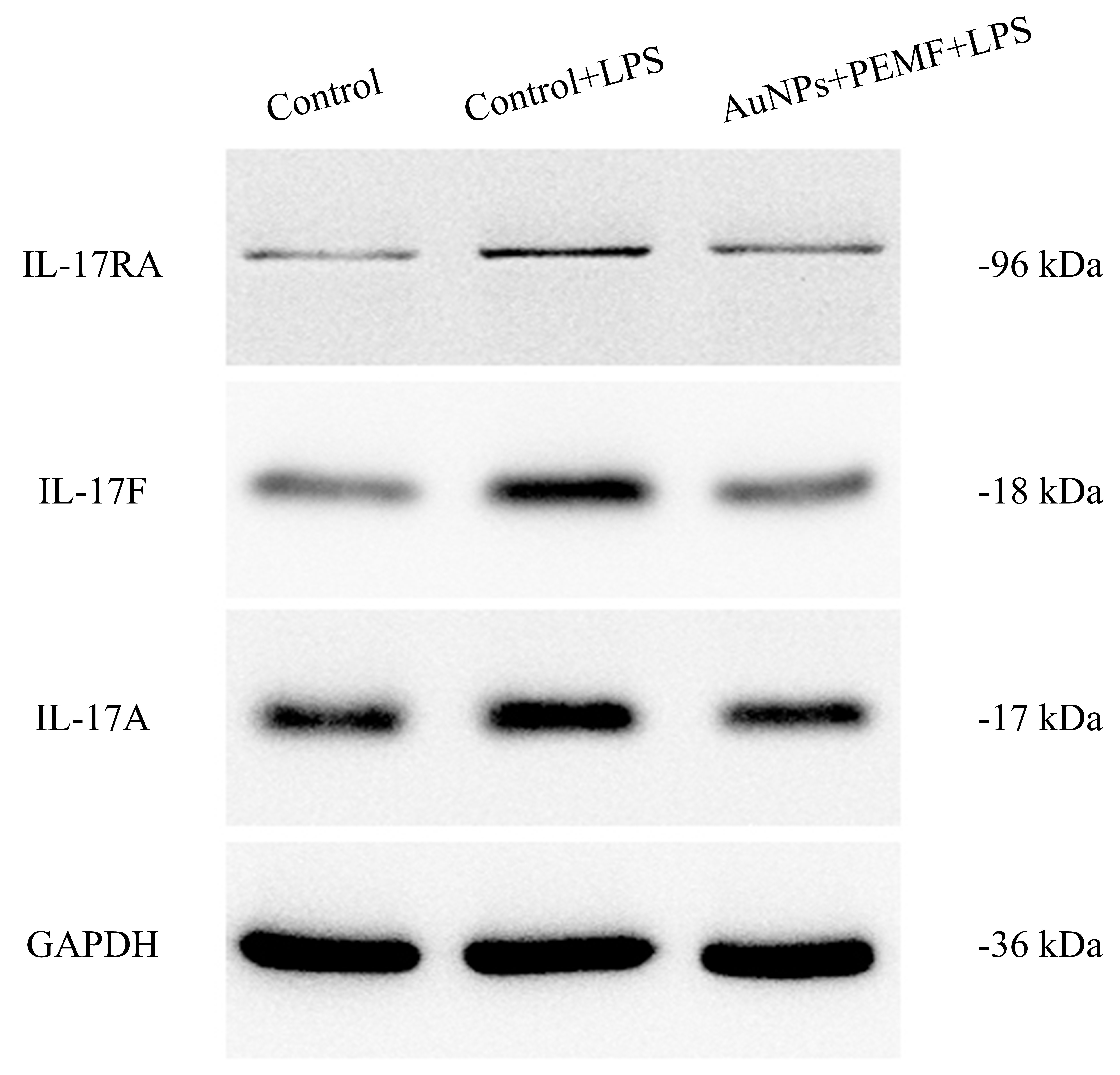


**Fig. S30.** WB analysis of IL-17A, IL-17F, and IL-17RA in femoral bone tissue.





**Fig. S31.** Quantification of IL-17A, IL-17F, and IL-17RA protein expression in femoral bone tissue. A one-way ANOVA was performed, with significance levels indicated as **p* < 0.05, ***p* < 0.01, and ****p* < 0.001.

**Table S1** Primer sequences for RT-qPCR.

| **Gene** | **Sequence** | |
| --- | --- | --- |
| Ndufa1 | Forward | ATGTGGTTCGAGATTCTCCCT |
|  | Reverse | TTTGTGGATGTACGCAGTGGA |
| Cox5b | Forward | GGAAGACCCTAATCTAGTCCCG |
|  | Reverse | GTTGGGGCATCGCTGACTC |
| Tomm20 | Forward | GCCCTCTTCATCGGGTACTG |
|  | Reverse | ACCAAGCTGTATCTCTTCAAGGA |
| Ptgs2 | Forward | TTCCAATCCATGTCAAAACCGT |
|  | Reverse | AGTCCGGGTACAGTCACACTT |
| IL-17A | Forward | CAGACTACCTCAACCGTTCCAC |
|  | Reverse | TCCAGCTTTCCCTCCGCATTGA |
| IL-17F | Forward | AACCAGGGCATTTCTGTCCCAC |
|  | Reverse | GGCATTGATGCAGCCTGAGTGT |
| IL-17RA | Forward | CTGTATGACCTGGAGGCTTTCTG |
|  | Reverse | CGAGTAGACGATCCAGACCTTC |
| Gapdh | Forward | AGGTCGGTGTGAACGGATTTG |
|  | Reverse | GGGGTCGTTGATGGCAACA |

**Table S2** Descriptions of the top 10 GO terms in BP, CC, and MF.

| **GO terms** | **Category** | **Description** |
| --- | --- | --- |
| GO:0042773 | BP | ATP synthesis coupled electron transport |
| GO:0042775 | BP | mitochondrial ATP synthesis coupled electron transport |
| GO:0022904 | BP | respiratory electron transport chain |
| GO:0045931 | BP | positive regulation of mitotic cell cycle |
| GO:0042254 | BP | ribosome biogenesis |
| GO:0006364 | BP | rRNA processing |
| GO:0009123 | BP | nucleoside monophosphate metabolic process |
| GO:0022613 | BP | ribonucleoprotein complex biogenesis |
| GO:0051347 | BP | positive regulation of transferase activity |
| GO:0007346 | BP | regulation of mitotic cell cycle |
| GO:0005747 | CC | mitochondrial respiratory chain complex I |
| GO:0030964 | CC | NADH dehydrogenase complex |
| GO:0045271 | CC | respiratory chain complex I |
| GO:0098803 | CC | respiratory chain complex |
| GO:0070469 | CC | respiratory chain |
| GO:0005746 | CC | mitochondrial respiratory chain |
| GO:0098800 | CC | inner mitochondrial membrane protein complex |
| GO:0044455 | CC | mitochondrial membrane part |
| GO:0098798 | CC | mitochondrial protein complex |
| GO:0005840 | CC | ribosome |
| GO:0004298 | MF | threonine-type endopeptidase activity |
| GO:0008137 | MF | NADH dehydrogenase (ubiquinone) activity |
| GO:0050136 | MF | NADH dehydrogenase (quinone) activity |
| GO:0003954 | MF | NADH dehydrogenase activity |
| GO:0043021 | MF | ribonucleoprotein complex binding |
| GO:0003735 | MF | structural constituent of ribosome |
| GO:0042393 | MF | histone binding |
| GO:0045296 | MF | cadherin binding |
| GO:0044389 | MF | ubiquitin-like protein ligase binding |
| GO:0031625 | MF | ubiquitin protein ligase binding |
